# Stabilizing bicontinuous particle-stabilized emulsions formed *via* solvent transfer-induced phase separation†

**DOI:** 10.1039/d4sm01213e

**Published:** 2025-01-07

**Authors:** Meyer T. Alting, Martin F. Haase

**Affiliations:** a Van ‘t Hoff Laboratory of Physical and Colloid Chemistry, Department of Chemistry, Debye Institute for Nanomaterials Science, Utrecht University Utrecht The Netherlands m.f.haase@uu.nl

## Abstract

Bicontinuous particle-stabilized emulsions (bijels) are unique soft materials that combine the bulk properties of two immiscible fluids into a single interconnected structure. This structure is achieved through the formation of two interwoven fluid networks, stabilized by an interfacial layer of colloidal particles. Bijels with submicron-scale domain networks can be synthesized *via* solvent transfer-induced phase separation (STrIPS). However, the fluid network structure in STrIPS-bijels tends to degrade over time, limiting their practical applications. In this study, we identify that the destabilization of STrIPS-bijels is driven by the exchange of matter between the bijel and the surrounding oil phase. Confocal laser scanning microscopy reveals that over time, aqueous components from the bijel dissolve into the surrounding oil, leading to an inward flow of oil into the bijel. This process disrupts the fluid bicontinuous structure within hours. To extend the stability of the bijel to several weeks, we explore strategies to reduce the dissolution of the aqueous phase and the inflow of oil. Specifically, we investigate the effects of the oil's chemical composition and properties, as well as modifications to the surface chemistry of the supporting glass substrates. Our results show that while the particle scaffold of STrIPS-bijels exhibits long-term stability, the maintenance of the fluid bicontinuous network depends on minimizing the loss of aqueous components. The enhanced control over the stability of the fluid bicontinuous structure developed in this work is critical for advancing STrIPS-bijels as functional materials for applications in catalysis, separations, and energy storage.

## Abstract 2

Bijels are non-equilibrium soft materials with potential applications in catalysis, membrane separations, and energy storage. In this study, we demonstrate the long-term stabilization of STrIPS-bijels, maintaining their structure for several weeks.

## Graphical abstract

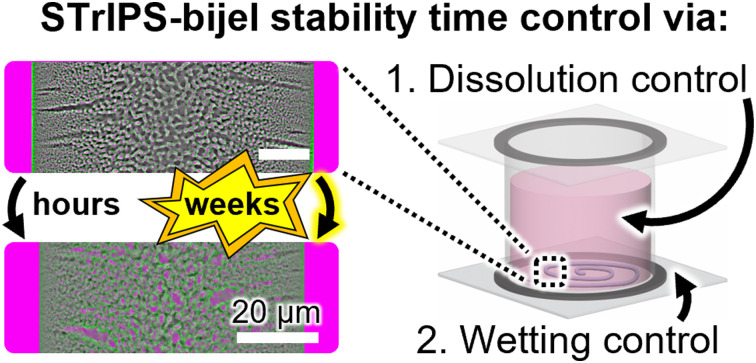

## 1. Introduction

Oil and water famously do not mix. Instead, when brought into contact, they form two separate liquid layers. Fine droplet dispersions of oil and water are known as emulsions and have many applications in food,^1,2^ cosmetic,^3^ pharmaceutical,^4^ and material sciences.^5–7^ Highly stable emulsions can be obtained with colloidal particles as stabilizers.^8–14^ The colloidal particles stabilize emulsions by forming protective coatings on the droplet's surfaces. However, compartmentalizing oil or water into a droplet geometry limits direct access to its content. The particle-stabilized droplet is an isolated entity due to the surrounding continuous liquid phase. Thus, the exchange of materials between the droplet and its surrounding environment is limited.

This limitation can be overcome with a ‘bijel’, a bicontinuous particle-stabilized emulsion.^15,16^ A perfect bijel does not contain any isolated droplets, because oil and water are fully intertwined into two continuous networks.^15^ These fluid networks are stabilized by a layer of colloidal particles at the interface. In fact, a bijel can be considered a tri-continuous material, because both liquid channel networks and the layer of colloidal particles between them are uninterrupted throughout the entire volume. Each of these three continuous components possesses unique physical and chemical properties, allowing the integration of three materials with distinct, adjustable characteristics into a single structure. These tri-continuous domains of the bijel facilitate the separate transport of mass, heat, and electricity, with potential applications for batteries,^17,18^ chemical reactors,^19,20^ separation membranes^21,22^ and biomaterials.^23,24^ But, for these applications to be realized the stability of the bijel structure must be ensured. Destabilization can result in disruptions of the structure, interrupting the transport of matter through the bijel.

Sanz *et al.* have investigated the stability for bijels fabricated *via* thermally induced phase separation (TIPS).^25^ They demonstrated that attractive particle-interactions enhance the stability of the bijel. The particle scaffold remains intact even when the oil and water phases are remixed or when the oil is exchanged.^26^ In contrast, bijels without these attractive interactions are more susceptible to destabilization.^27^ Bijel stability can be further improved through droplet bridging, allowing for the formation of stable bijels with domain sizes reaching hundreds of micrometers.^28^ Such large domain bijels have also been stabilized through direct mixing by density matching the oil and water phases.^29^ While these results have provided valuable insights into the stability of bijels made *via* TIPS and direct mixing, the stability of bijels made *via* solvent transfer induced phase separation (STrIPS) have not been sufficiently addressed in the literature yet.^30^

STrIPS enables the scalable production of bijel fibers, sheets and microparticles through continuous flow synthesis. Additionally, STrIPS accommodates a wide variety of immiscible liquids for bijel fabrication, offering remarkable versatility.^31–33^ This flexibility facilitates the creation of bijels with submicron domains, because nanoparticles can be used as stabilizers.^34–36^ Unlike TIPS-bijels, which are typically confined by container walls, STrIPS-bijels are immersed in a continuous liquid phase.^37^ This allows the STrIPS-bijel to interact with the surrounding liquid, introducing additional factors that influence its stability.

Here, we hypothesize that the exchange of matter between the STrIPS-bijel and the surrounding liquid affects the stability of the bicontinuous fluid networks. To test this hypothesis, we synthesize STrIPS-bijel fibers in a continuous oil phase and investigate their stability by confocal laser scanning microscopy. We find that enriching the oil phase with aqueous components and increasing the oil's viscosity significantly extended bijel stability from hours to several weeks. Additionally, hydrophobization of the support glass surface and preventing water evaporation contributes to the long term stability. While previous reports have extensively explored the formation of bijel structures *via* STrIPS,^22,38–40^ this work offers crucial insights into the stability of STrIPS-bijels, essential for realizing their application potential.

## 2. Experimental

### 2.1 Materials

All chemicals were used as received. Toluene (99+%, extra pure), *n*-dodecane (99%), diethyl phthalate (DEP, 99%), glycerol (99+%, synthetic) and hydrochloric acid (37%) were purchased from Thermo Scientific. 1-Propanol (≥99.5%), hexadecyl-trimethylammonium bromide (CTAB, ≥99%), sodium chloride (for analysis), light mineral oil (density 0.84 g mL^−1^) and Nile red (for microscopy) were received from Sigma-Aldrich. Silica nanoparticles (Ludox® TMA, batch number 1003481587, particle diameter 29 nm) were purchased from Grace GmbH. *n*-Hexane (99% HPLC) was received from Biosolve BV. Octadecyl trichlorosilane (OTS, 94.3%) was purchased from Santa Cruz Biotechnology, Inc. Water used in all experiments was ultrapure MilliQ produced by a Rephile Genie U2 system with a resistivity of 18.2 MΩ cm.

### 2.2 Preparation of bijel precursor

50 mL of 34 wt% Ludox TMA dispersion (batch number 1003481587) is brought to pH 2.0 by adding 1 M aqueous HCl for two days to equilibrate the particles with the acidic environment. It is concentrated from 34 wt% to 45 wt% in a rotary evaporator (Heidolph Instruments) at 60 °C and 140 mbar and reacidified to pH 2.0. The dispersion is transferred to a dialysis bag (Spectra/Por MWCO 12–14 kD) and placed in a beaker containing 2.0 L MilliQ water at pH 2.0 containing 50 mM NaCl overnight. The dispersion is centrifuged at 3000 g for 10 minutes (Allegra X-12R, Beckman Coulter) to remove any particle aggregates. We determine the weight percentage of the centrifuged dispersion to 45 wt% by evaporating 2 mL of the supernatant and subsequent dry mass determination. Then, the dispersion was diluted to 40 wt% by adding MilliQ water at pH 2.0 containing 50 mM NaCl. Alternative to this Ludox TMA particle dispersion preparation, a more rapid dispersion preparation also enables the formation of bijel structures, as described previously.^35^ However, the stability results obtain here have employed the procedure described above.

The bijel precursor mixture is composed of four liquids with volume fractions *φ*_DEP_ = 0.078, *φ*_H_2_O_ = 0.435, *φ*_1-propanol_ = 0.381 and *φ*_glycerol_ = 0.106. Additionally, Ludox TMA nanoparticles with a weight percentage of 23 wt% and a CTAB concentration of 29.9 mM with the total mass/volume of the four liquids as a basis. To prepare 50 mL of this precursor, the following liquids were mixed: 3.50 mL of DEP, 6.67 mL of 200 mM CTAB in 1-propanol, 11.88 mL of a 50 wt% glycerol in 1-propanol, 2.95 mL of 1-propanol and 25.00 mL of the 40 wt% Ludox TMA dispersion prepared as discussed above. Additional details about the preparation of these bijel precursor mixtures can be found in ref. 34 and 35.

### 2.3 Enrichment of oils by water and glycerol

Oils were enriched by either water or both water and glycerol by vigorously mixing 80 mL of oil with 10 mL of either MilliQ water at pH 2.0 or 23 wt% glycerol in MilliQ water at pH 2.0 using a magnetic stirrer for 50 hours at room temperature in a closed vial. The mixture was stopped mixing for at least 1 hour to enable phase-separation before using the top-layers of the oil-phase.

### 2.4 Water-measurement in oils

The concentration of free water in the aqueous enriched oils is determined using a handheld water-in-oil test fabricated by CM Technologies GmbH. In brief, 30 mL of an oil is loaded into an sealed stainless steel container filled with anhydrous organics and a paste of calcium hydride. Upon reaction in the closed container, all water present in the oil is converted into hydrogen gas that generates a pressure, which is proportional to a particular concentration of water present in the oil.

### 2.5 Bijel fiber extrusion and storage

In brief, a fiber extrusion device consisting of a 1 cm long borosilicate glass capillary (inner diameter 50 μm, outer diameter 80 μm, CM Scientific, CV0508) is placed in a disposable 1 mL pipette tip and glued using Norland adhesive 81. Around 25 μL of precursor mixture is loaded into the space above the inlet of the glass capillary. The outlet of the capillary is submerged into 4 mL water-enriched toluene, contained within a glass container (diameter ∼2 cm) attached onto a glass coverslip (22 × 22 × 0.1 mm, VWR or Epredia) with epoxy glue (Liqui Moly 6183). Applying an air pressure of 4 bar above the precursor mixtures enables the extrusion of a 80–120 μm-diameter fiber. Approximately 5 cm of fiber is extruded in toluene. A more detailed description of this procedure can be found in ref. 35.

Then, the toluene is replaced by another oil (*n*-hexane, toluene, *n*-dodecane, light mineral oil or mixtures of light mineral oil containing 9, 23 or 47 wt% *n*-dodecane; all oils used as either as-received, water-enriched or water + glycerol enriched) containing Nile red *via* three consecutive washing steps of 2 mL of oils. The last washing step contains the Nile red in the oils. The bijels were stored at temperatures between 19 and 21 °C.

### 2.6 Confocal laser scanning microscopy analysis

The fibers are analyzed by inverted confocal laser scanning microscopy (CLSM, Stellaris 5, Leica Microsystems) equipped with a water-immersion objective with magnification of 63×, numerical aperture of 1.2 and a resolution of 250–300 nm (Rayleigh criterion). Bijels are stored in oil saturated with Nile red as fluorescent dye. In the bijel, Nile red remains dissolved in oil and adsorbs on CTAB-modified Ludox TMA particles. Two separate lasers of 488 and 561 nm are used to excite, respectively, the dissolved and adsorbed Nile red. Dissolved Nile red emits a green fluorescence detected at 500–550 nm, corresponding to the oil-phase in and surroundings of the bijel. Adsorbed Nile red emits an orange-red fluorescence detected at 600–700 nm, corresponding to the position of the particle network in the bijel. Images at the equator and below are acquired at different time intervals. Our method has the advantage that no continuous imaging is required. Additional details about acquiring the fluorescence emission of Nile red in bijels can be found in ref. 34 and 35.

### 2.7 Confocal data processing

Images acquired by CLSM have been processed using ImageJ Fiji software. Due to refractive index mismatch, the contrast of the images varied per sample. A bandpass filter has been applied to both images and this data has been used as alternative towards common brightness/contrast adjustment procedures (see ESI,† S1).

### 2.8 Glass container hydrophobization

Glass containers are treated by a 3 vol% OTS solution in mineral oil overnight and rinsed with *n*-hexane.

### 2.9 Viscosity measurements

Viscosities of mixtures between both *n*-dodecane and mineral oil have been measured using a MCR300 Paar Physica rheometer equipped with the CP50-1 Cone Plate system. The viscosity is measured at 20.00 °C at shear rates between 10 to 300 s^−1^ in 30 steps for 6 seconds each.

## 3. Results and discussion

### 3.1 Experimental system to investigate bijel stability

**Fig. 1:**
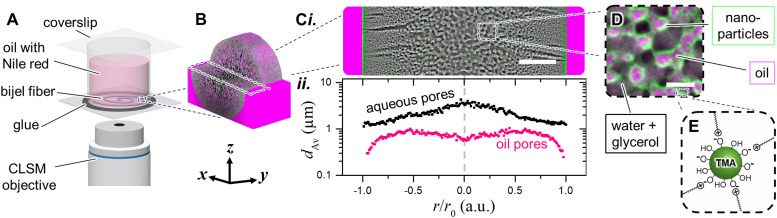
Experimental setup and bijel system. (A) Glass container filled with oil containing bijel fiber. (B) 3D confocal reconstruction of bijel fiber stored in *n*-hexane with indication of equator. (C(i)) Confocal image of equator of a bijel fiber segment stored in *n*-hexane with oil (magenta), aqueous (black) and particle-network (green) processed with a bandpass filter. Scale bar is 20 μm. (C(ii)) Average pore size (*d*_Av_) as a function of the normalized radial position. (D) Magnified CLSM image of oil- and aqueous pores. Scale bar is 3 μm. (E) Schematic drawing of CTAB-modified Ludox TMA silica NPs.

Bijels are synthesized *via* STrIPS by means of a single channel flow device.^35^ In this report, the fibers have a radius *r*_0_ of 60 ± 10 μm and a total volume of 0.6 μL. They are surrounded by a larger volume of several milliliters of oil. Due to their higher density, they rest on a glass surface at the bottom of a container. The container is closed by placing a coverslip on top. Below the container is an objective of a confocal laser scanning microscope (CLSM), as schematically depicted in Fig. 1A.

Fig. 1B shows a 3-dimensional CLSM image of a cylindrical bijel fiber. In this study, we focus on the equatorial cross-section of this fiber, shown in Fig. 1C(i). This image reveals the interwoven networks of oil (magenta), and aqueous phase (black), as well as the nanoparticles (green). The magenta and green color represent false coloring of the 2 different fluorescence signals of Nile red in the adsorbed and the dissolved state.^34,35^ At normalized radial positions *r*/*r*_0_ > 1.0, the CLSM image shows the bulk oil phase. The interior of the bijel fiber is visible for *r*/*r*_0_ < 1.0, showing two distinct types of pores. First, radially aligned macrovoids filled with aqueous phase are visible. Second, interwoven oil/aqueous pores with a radial size gradient are present. This gradient is quantified in Fig. 1C(ii), which plots the average pore sizes (*d*_Av_) of both oil- and aqueous pores. The oil-pores range from 0.3 to 1.0 μm in size, whereas the aqueous network has an increasing pore size gradient from 1.0 μm at *r*/*r*_0_ = 1.0 to 5.0 μm at *r*/*r*_0_ = 0. The pore size gradient evolves due to diffusion phenomena, as investigated previously.^34^ These pores size distributions confirm that the resolution of our CLSM images is sufficient to distinguish the submicron oil-domains and micron-sized aqueous domains.

Fig. 1D further magnifies the CLSM image of the interwoven oil/aqueous pores. The black aqueous pores contain a mixture of water and glycerol. Glycerol has been added to the precursor mixture to match the refractive index (RI) of aqueous and oil phase.^35^ RI-matching reduces light scattering and enhances CLSM image sharpness. Fig. 1D also shows a distinct green layer between the oil and aqueous pores. This layer consists of interfacially attached Ludox TMA silica nanoparticles. The nanoparticles are rendered interfacially active by adsorption of cetyltrimethylammonium bromide (CTAB), as schematically depicted in Fig. 1E. The magenta oil phase in Fig. 1D is composed of *n*-hexane. In this study, we will vary the chemical composition of the oil network to investigate bijel stability.

**Fig. 2:**
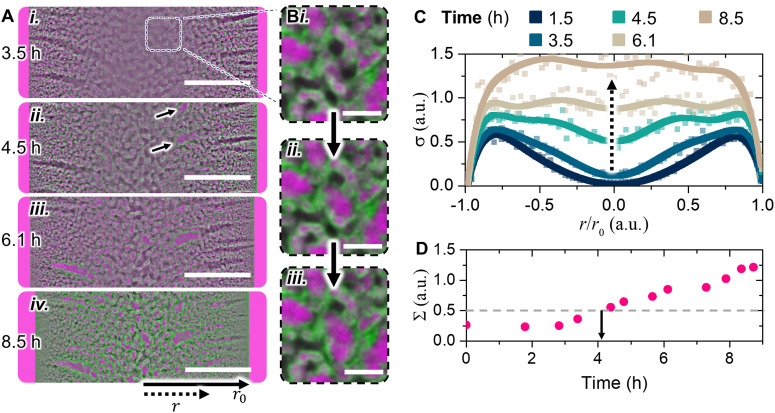
Stability of bijel fiber stored in *n*-dodecane. (A) Confocal time series of equator of bijel fiber stored in *n*-dodecane with Nile red (processed with a bandpass filter in ImageJ). Scale bar = 20 μm. (B) Magnification of oil-expansion near the center of the fiber. Time step between images is 2 minutes, scale bar = 4 μm. (C) Area ratio *σ*(*r*/*r*_0_) = *A*_oil_/*A*_aqueous_ against normalized radial position *r*/*r*_0_ at different times. Square symbols correspond to measured data, lines are guides-to-the-eye. (D) Overall bijel area ratio 
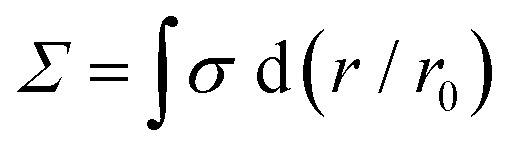
 at fiber equator over time.

We first study the bijel stability in an oil phase composed of *n*-dodecane. Fig. 2A(i) shows a CLSM image at the equator of a bijel fiber stored in *n*-dodecane after 3.5 hours after bijel synthesis. The center appears less sharp compared to the bijel in *n*-hexane (Fig. 1C(i)), implying a poorer RI-matching with *n*-dodecane. After 3.5 hours the interior appears similar to the initial structure directly after synthesis (see video S1, ESI†). However, between 3.5 and 4.5 hours, the interior begins to change as shown in Fig. 2A(ii). The CLSM image at the center becomes sharper. Moreover, larger oil domains appear as highlighted by arrows in Fig. 2A(ii).

Fig. 2B magnifies the time evolution of a larger oil domain located in the dashed box of Fig. 2A(i). This evolution starts after four hours and takes place at different locations in the fiber. Fig. 2B(i) shows an example of an original network structure after four hours. After 2 additional minutes, the abrupt growth of an oil-domain displaces the aqueous domain as shown in Fig. 2B(ii). During this growth, the particle layer moves along with the oil–water interface, preventing coalescence with adjacent oil-domains. This expansion continuous after two more minutes and eventually (after several hours) the aqueous network is completely displaced by oil domains. Thus, the oil domains expands at the expense of the aqueous domains while keeping the particles attached to the liquid–liquid interface.

This type of oil domain expansion occurs at various locations in the fiber over the course of 8.5 hours, as shown in Fig. 2A(iii) and (iv). Simultaneously, *r*_0_ of the fiber decreases from 73 to 67 μm. Furthermore, the CLSM image sharpness improves further. Notably, the overall particle scaffold structure remains intact. After this qualitative assessment, we quantitate the bijel destabilization for our analysis in the following.

To account for the growth of the oil domains in the fiber, we measure the areas of oil and aqueous domains in the bijel. The areas are determined from the fluorescence signals in the CLSM images at different times in one cross-sectional plane of the fiber (see ESI,† S2). These measurements provide the area-based ratio of the oil- and aqueous networks, denoted as *A*_oil_/*A*_aqueous_. By analyzing *A*_oil_/*A*_aqueous_ at various radial positions, expressed as *σ*(*r*/*r*_0_) = *A*_oil_/*A*_aqueous_, the local growth of the oil domains can be further characterized. However, this 2-dimensional area-analysis may not completely represent volumetric changes in bijels as no 3-dimensional CLSM images have been recorded due to practical limitations (see ESI,† S3).

Interestingly, the 2-dimensional analysis reveals that the oil domain growth begins in the center of the fiber. Fig. 2C plots *σ* over *r*/*r*_0_ (individual plots in ESI,† S4). After 1.5 hours, the radial profile of *σ* shows two maxima at *r*/*r*_0_ = ±0.8 and a minimum at *r*/*r*_0_ = 0. This profile indicates that the aqueous content decreases as *r*/*r*_0_ increases. After 4.5 hours, *σ* increases primarily at *r*/*r*_0_ = 0 with a spread of ±0.05. As time proceeds up to 8.5 hours, *σ* also increases for 0 < *r*/*r*_0_ < 0.75 with an overall spreading of ±0.1. These observations suggest that the growth of the oil domains starts in the center of the fiber and proceeds across the full width at later stages with some local differences. This local analysis provides the input for the overall stability assessment of the bijel.

To quantify the overall bijel stability, we determine the oil to aqueous area ratio for the entire fiber cross-section using 
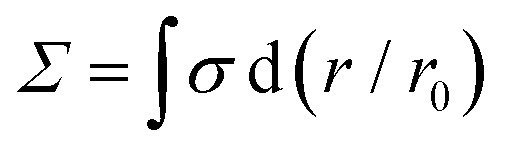
. To assign *Σ* as stability criterion, we define STrIPS-bijel stability as the time required for the oil phase to displace the aqueous phase within the bijel. Evaluation of *Σ* at different times enables us to track the kinetics of the oil/aqueous rearrangement and compare different bijel destabilization profiles (see ESI,† S9). While a limitation of *Σ* is its inability to provide precise volumetric measurements of the oil and aqueous phases (see ESI,† S3), it nonetheless allows for the quantitative detection of distinct bijel destabilization mechanisms.

Evaluating *Σ* at the equator of as-synthesized STrIPS-bijel fibers gives a specific value of 0.25 ± 0.05. Fig. 2D plots *Σ* over time for the bijel fiber in *n*-dodecane. It shows that *Σ* remains constant around 0.25 ± 0.05 for 3 hours and increases to 1.2 ± 0.1 within 9 hours after bijel synthesis (see ESI,† S2). This increase can be attributed to displacement of the aqueous phase by oil, and reduction of the fiber diameter.

We define *Σ* = 0.5 as the arbitrary threshold value for quantifying the onset of bijel destabilization. Within 3 hours, *Σ* typically increases from 0.25 to values around 0.4 solely due to shrinkage of the fiber diameter (see ESI,† S5 and Video S1). From 4 hours on, *Σ* exceeds 0.5 due to filling of the water cavities in the bijel with oil. Using *Σ* = 0.5 ± 0.05 allows us to pinpoint this onset of oil expansion within the bijel within 15% uncertainty (see ESI,† S6) while the time-dependent evolution of *Σ* provides a means to compare the destabilization kinetics across different experimental conditions. For example, bijel fibers stored in *n*-dodecane have a stability period of approximately 4 hours, as plotted with the dashed line in Fig. 2D.

What is the cause for the destabilization of the bijel network? Fig. 2A shows that the oil domains grow into the aqueous domains. The growth begins in the radial channels and near the center of the fiber where the largest aqueous domains are located. Additionally, the fiber diameter decreases over time and the RI-matching improves. This suggests that the aqueous phase leaves the bijel interior. We hypothesize two possible mechanisms for the leaving of the aqueous phase: (I) dissolution into the surrounding oil, or (II) outflow onto the glass surface. In the next section, we investigate the effect of both possible mechanisms on the bijel stability.

### 3.2 Mechanisms of aqueous phase departure out of bijel

Dissolution of water and glycerol into the surrounding oil is plausible because the volume of the *n*-dodecane is more than 1000 times larger than the volume of the bijel fibers. The solubility of water and glycerol in *n*-dodecane at 293 K are, respectively, 50–200 and 1600 μL L^−1^ (see ESI,† S7).^41–43^ Based on the small volume of the fibers compared to the bulk oil, all water and glycerol can dissolve out of the bijel into the surrounding *n*-dodecane (see ESI,† S7). To test dissolution of the aqueous phase, we vary the concentration of water and glycerol in *n*-dodecane.

The concentration of water in *n*-dodecane is determined by measuring the evolution of hydrogen gas upon reaction with calcium hydride. This measurement reveals that as-received *n*-dodecane contains ∼85 μL L^−1^ water, slightly below the maximum solubility of water in *n*-dodecane.^42^ Thus, we increase the water concentration further to 110 ± 5 μL L^−1^ by vigorous agitation of a mixture of *n*-dodecane and water (see ESI,† S8). Since this concentration is still below the maximum solubility, we refer to this as water-enriched rather than water saturated *n*-dodecane. We test the stability of bijel fibers stored in water-enriched *n*-dodecane in the following.

**Fig. 3:**
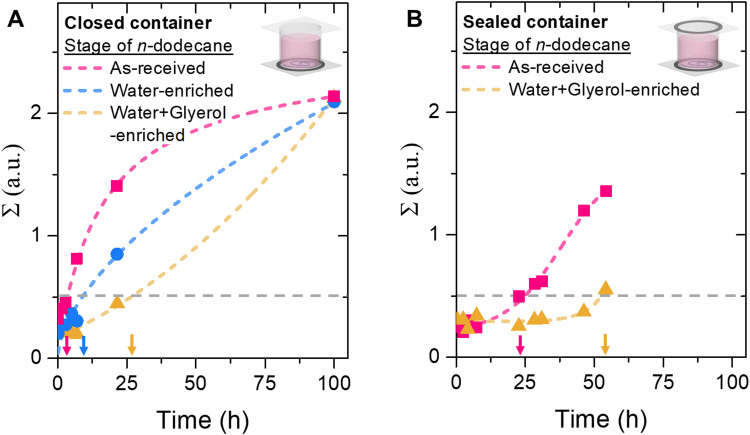
Effect of aqueous enrichment of the bulk oil on bijel stability. (A) *Σ* against time for a closed container for bijels stored i*n n*-dodecane used as-received or enriched by water alone or both water and glycerol. (B) *Σ* against time for bijels stored in a sealed container in as-received and water + glycerol enriched *n*-dodecane. Lines are drawn to guide the eye.

Fig. 3A plots *Σ* over time for bijels stored in as-received and in water-enriched *n*-dodecane. This plot shows that the increase of *Σ* slows down as the *n*-dodecane is enriched by water. The stability period, defined as the time when *Σ* < 0.5, increases from 4 to nearly 10 hours. Is it possible to prolong the stability range further? To test this, we additionally enrich *n*-dodecane with both water and glycerol.

Interestingly, the water concentration in *n*-dodecane enriched by both water and glycerol of 100 ± 5 μL L^−1^ is slightly lower than in *n*-dodecane solely enriched by water. Due to instrumental limitations the glycerol concentration cannot be measured in this work. Nevertheless, the simultaneous enriching by both water and glycerol has a significant effect on the bijel stability as shown Fig. 3A. The increase of *Σ* slows down further, and the stability period extends to over 25 hours. These observations support the hypothesis that both water and glycerol dissolve from the bijel to *n*-dodecane. Increasing the concentration of both aqueous components slows down the dissolution, enhancing the stability period over five times.

However, even in water + glycerol enriched *n*-dodecane, the aqueous network leaves the bijel. A possible cause can be the evaporation of water from the *n*-dodecane. Fig. 3B shows that hermitically sealing the container further enhances the stability period for both enriched and as-received *n*-dodecane. Water + glycerol enriched *n*-dodecane displays a stability period of over 50 hours, suggesting that water evaporation is significant (see ESI,† S9 and S10). Interestingly, for both closed and sealed bijel storage containers, water condensation has been observed on the container wall. The condensation indicates saturation of the air with water vapor, which limits further evaporation of water (see ESI,† S10). Thus, enriching the oil with both water and glycerol are essential for bijel stability, but additionally the evaporation of water needs to be prevented.

Besides the dissolution of water and glycerol into *n*-dodecane, the aqueous phase can also flow out of the bijel onto the supporting glass surface. To test whether the aqueous phase can flow on the glass surface, we vary the hydrophobicity of the glass. To this end, we compare bijel stability on a bare glass slide and a octadecyl trichlorosilane (OTS)-treated glass slide. The OTS-treatment increases the contact angle of a water droplet in air from 45° to 150° (see ESI,† S11). Indeed, this OTS treatment affects the stability of the bijel as discussed next.

**Fig. 4:**
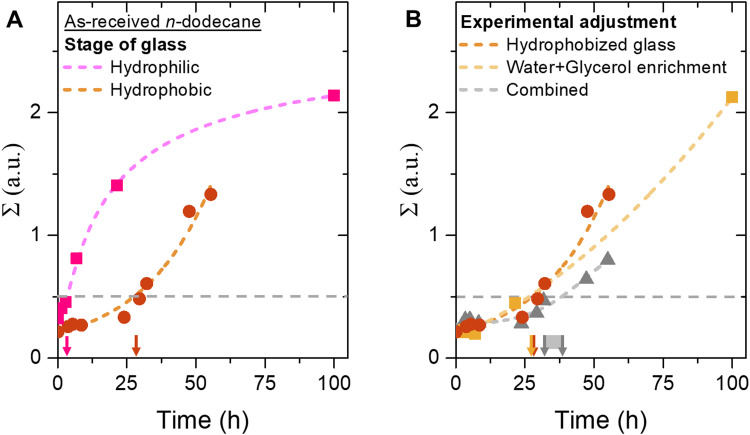
Effect of glass surface wettability on bijel stability. (A) Plot of *Σ* against time for bijel fibers supported on either a hydrophilic (squares) or hydrophobic (circles) glass stored in as-received *n*-dodecane. (B) Comparison of *Σ* over time for bijel fibers stored in hydrophobized glass (brown), water + glycerol enriched *n*-dodecane (yellow), and combined hydrophobization + enrichment (grey). Lines are drawn to guide the eye.

Fig. 4A shows the change in *Σ* over time for bijels stored in as-received (unenriched) *n*-dodecane, with both hydrophilic and hydrophobic unsealed containers. This plot shows that the increase of *Σ* slows down significantly as the bijel is supported on a hydrophobic substrate. On a hydrophobic substrate, *Σ* exhibits a concave profile, starting with a nearly constant value followed by a steep increase. In contrast, on a hydrophilic glass surface, *Σ* displays a convex profile, characterized by an initial steep rise that gradually flattens. These distinct behaviors suggest that different destabilization mechanisms are at play. In addition, the bijel stability period increases from 4 hours to over 1 day. This result supports the hypothesis that the aqueous phase can leave the bijel by flowing on the glass surface. Weakening the interactions between the glass substrate and the aqueous phase upon hydrophobization enhance bijel stability over five times.

Interestingly, this five-fold stability increase mirrors the prolongation previously observed with water and glycerol enrichment of *n*-dodecane. It seems that the aqueous phase exits the bijel through two mechanisms: dissolution into the oil and wetting-driven flow on the glass substrate, both occurring to a similar extent.

Will the stability period of the bijel prolong beyond 1 day if both the *n*-dodecane is enriched and the glass is hydrophobized? Fig. 4B plots the change in *Σ* against time for bijel fibers where *n*-dodecane enrichment has been combined with glass hydrophobization in an unsealed container. The plot shows that the changes in *Σ* for the combined adjustments of hydrophobization and enriching is similarly shaped to those observed with either adjustment alone. The similarity of the curves suggests that the kinetics of oil/aqueous rearrangement in bijels are comparable under both conditions. In contrast, the combination of hydrophobzation and enrichment extends the bijel stability period to 30–35 hours. This stability improvement is slightly longer than the 25 hours stability observed for either enriching or hydrophobization alone. Therefore, we conclude that the bijel stability period is not simply determined by summing the bijel stability for enriching and hydrophobization. The precise mechanism by which the combination of wetting and dissolution governs bijel stability remains unclear in this study. For example, despite the hydrophobization, glycerol can interact with the hydrophobic coating on the glass.^44,45^ This can increase the wettability of the aqueous phase on the glass surface (see ESI,† video S2). As a result, wetting effects can still play a significant role for bijel stability.

To summarize this section: Bijel stability can be extended to over 35 hours by minimizing aqueous dissolution into the surrounding oil and reducing aqueous wetting on the glass surface. During destabilization, water and glycerol exit the bijel, being displaced by particle stabilized oil domains. For this process to occur, oil must enter the fiber through inward flow. Can increasing the oil's viscosity slow this inward flow and additionally decrease water and glycerol dissolution? We explore this question in the next section.

### 3.3 Viscosity effect on bijel stability

**Fig. 5:**
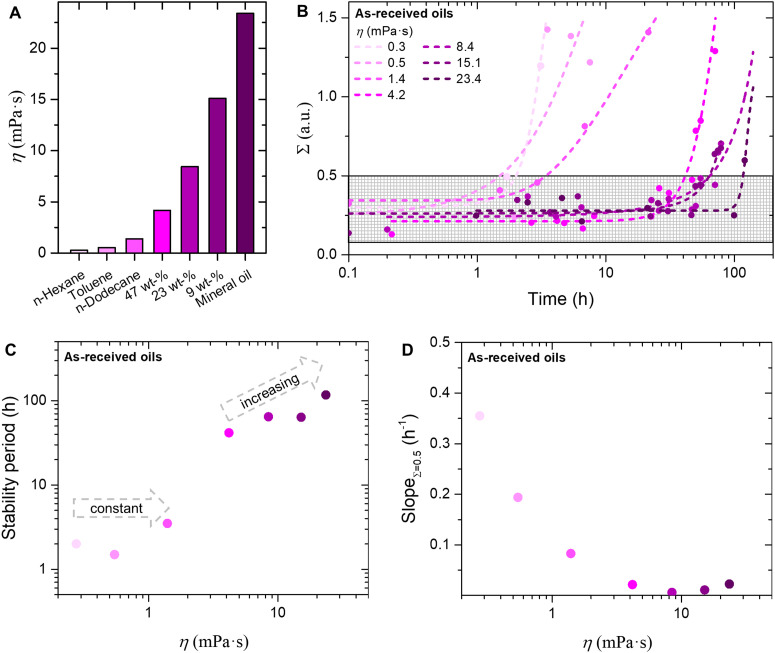
Effect of viscosity on bijel stability. (A) Comparison of the oil viscosities. (B) *Σ* plotted on a logarithmic time axis. Lines are drawn to guide the eye. The dashed gray rectangle highlights the region where bijels are stable. (C) Stability period against viscosity for bijels stored in as-received oils. (D) Slope of *Σ*(*t*) at *Σ* = 0.5 against viscosity.

To assess the effect of oil viscosity on bijel stability, we store fibers in oils with variable viscosity (*η*) ranging from 0.3 to 24 mPa s, as shown in Fig. 5A. Toluene and *n*-hexane are less viscous than *n*-dodecane (*η*_*n*-dodecane_ = 1.395 mPa s), light mineral oil is more viscous. Intermediate *η* are obtained by mixing *n*-dodecane with light mineral oil (9, 23 or 47 wt% *n*-dodecane in light mineral oil, see ESI,† S12). These oils have neither been enriched by water nor glycerol. Moreover, the containers storing the fibers have untreated, hydrophilic glass surfaces. The containers are closed, and not sealed.

Fig. 5B plots *Σ* for several *η* on a logaritmic time scale (individual plots shown in ESI,† S13). Initially, *Σ* varies around 0.25 irrespective of the storage oil, similar as found for *n*-dodecane before. This ratio remains constant around 0.25 and increases at different times depending on the oil. The grey box displays the stability period of the bijel before *Σ* reaches 0.5. Fig. 5B shows that varying *η* affects both the bijel stability period, and the rate of bijel destabilization. Both observations are discussed separately in the following.

The bijel stability period (duration until *Σ* equals 0.5) is plotted against *η* in Fig. 5C. Storing bijels in oils with *η* between 0.3 and 1.4 mPa s results in stabilities around 2 hours, showing no clear trend (see ESI,† S14). However, increasing *η* from 1.4 to 4.2 mPa s extends the stability from 2 hours to 2 days. Increasing *η* further to 23.4 mPa s increases the stability further to 4 days. Thus, the bijel stability period extends drastically by increasing *η* from 1.4 mPa s to 4.2 mPa s.

The rate of bijel destabilization is defined as the slope of the *Σ versus* time curve at the point where *Σ* equals 0.5 (Slope_*Σ*=0.5_, see ESI,† S15). Fig. 5D plots Slope_*Σ*=0.5_ against *η*. This plot shows that Slope_*Σ*=0.5_ gradually decreases from 0.35 to 0.02 h^−1^ by increasing *η* from 0.03 to 8.4 mPa s, respectively. The slope determination for *η* beyond 10 mPa s does not allow for accurate analysis, but Slope_*Σ*=0.5_ remains low around 0.03 h^−1^. Therefore, increasing the oil viscosity gradually slows down the rate of bijel destabilization.

**Fig. 6:**
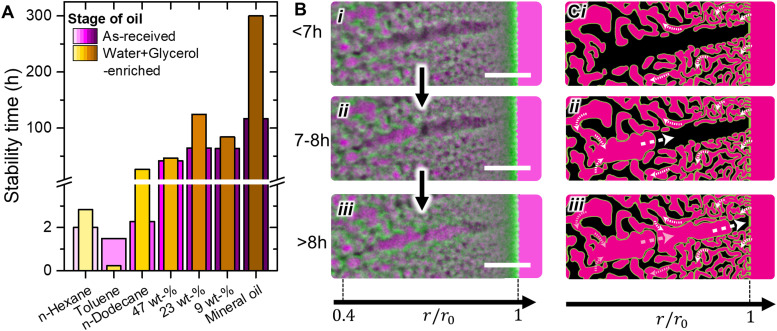
Enriching of oils and oil penetration during bijel destabilization. (A) Stability time of bijels stored in as-received and water + glycerol enriched oils. (B) CLSM images of oil expansion into radial channel. Scale bar is 5 μm. (C) Schematic depiction of proposed inward flow of oil into bijel interior.

Increasing *η* can slow down bijel destabilization *via* two potential mechanisms: (I) reducing the rate of dissolution and diffusion of water/glycerol out of the bijel due to a reduction of the diffusion coefficient, (see Stokes–Einstein–Sutherland equation), and (II) increasing the resistance for oil to flow into the bijel. To probe the diffusion of water and glycerol, we once again enrich the various oils. Bijel stability is tested with these enriched oils and the measured stability period is plotted in Fig. 6A (see ESI,† S16 for changes of *Σ* over time).

Fig. 6A shows the stability period of bijels stored in water and glycerol enriched oils. Enriching toluene shortens the stability period from 1.5 to 0.25 hours. In contrast, enriching alkanes and their mixtures significantly increases bijel stability. The different behavior for toluene and alkanes may be due to polarity differences (see ESI,† S17). Interestingly, the stability period increase differs among the alkanes. For example, for 23 wt% *n*-dodecane in mineral oil, the stability increases from 65 to 125 hours, while for 47 wt% it increases only from 42 to 46 hours upon enriching. Remarkably, for light mineral oil alone, enriching increases the stability from 117 to 300 hours. Thus, the stability period increase is not directly proportional to *η* upon enrichment. Although the various alkanes have similar polarity and water solubilities (see ESI,† S17), it is possible that our experimental procedure has not resulted in the same enrichment by water and glycerol for these samples (see ESI,† S8). Nevertheless, enriching extends the stability period of the bijels stored in alkanes and their mixtures, confirming that the dissolution of water and glycerol drives bijel destabilization. Crucially, as the aqueous phase leaves the bijel, the resulting volumetric loss must be compensated. Consequently, oil must flow into the fiber, as discussed next.

To visualize the inflow of oil and the expansion of oil domains during the destabilization, the aqueous displacement in a radial channel is imaged *via* a CLSM in Fig. 6B. The CLSM time series is recorded for *n*-dodecane as the surrounding oil (similar behaviors are observed for all alkanes investigated here). Fig. 6B(i) shows a radial channel, initially filled with the aqueous phase. Fig. 6B(ii) and (iii) show the displacement of the aqueous phase by *n*-dodecane within several hours. During this displacement, the particle layer remained attached to the liquid–liquid interface similar as discussed in Fig. 2B. Notably, the expansion of *n*-dodecane into the radial channel occurs in positive radial direction towards the outer fiber surface. In agreement with Fig. 6B, Fig. 2C shows that the oil to aqueous ratio initially increases near the center of the fiber. During this initial increase, the aqueous domains near the outer surface of the fiber remain stable. We speculate that differences in mechanical strength within the particle scaffold cause the oil to expand near the center of the fiber. The structure near the outer surface of the fiber is composed of a smaller and dense scaffold, providing higher mechanical strength. In contrast, the structure near the center is composed of a larger and less dense scaffold with weaker mechanically properties.^27,46^ This interpretation offers a potential explanation why *n*-dodecane breaches through the weakest point in the center of the bijel scaffold.

The radial expansion of oil domains from the center of the fiber suggests that *n*-dodecane flows from the surrounding bulk phase into the fiber. We speculate that *n*-dodecane enters through pores on the fiber's outer surface and flows inward through the oil channel network, as schematically depicted in Fig. 6C.^38^ This inward penetration of oil through the submicron oil channels requires overcoming a significant flow resistance, which must be overcome to compensate for the volumetric loss caused by the removal of water and glycerol. The more viscous the oil, the higher the flow resistance. Consequently, the removal of water and glycerol may be delayed as the viscous oil struggles to penetrate the fiber. This interpretation could explain the longer stability times and slower destabilization rates with increasing oil viscosity (Fig. 5C and D). However, further research is needed to fully validate this proposed mechanism.

Before concluding, we discuss the stability of the particle scaffold upon exchange of the oil phase. Previous reports showed that attractive interactions *e.g.* van der Waals forces, between the interfacially jammed particles can provide structural stability against liquid exchange.^25,27^ Previous work has also shown that CTAB-functionalized particles provide stability to change the oil-phase for STrIPS-bijels.^34^ Exchange of the oil, though, may affect the concentration of physically adsorbed CTAB cations and liquid–liquid interfacial tension. As the oils used in this study have similar polarities and interfacial tensions with water (see ESI,† S17), no significant changes in CTAB adsorption are expected. Neither the particle contact angle is expected to change significantly. The particles will remain attached to the liquid–liquid interface and are unlikely to impede structural changes upon the exchange of oil. However, more research is needed to study the strength of the particle scaffold for various oil compositions.

## 4. Conclusions

In summary, this manuscript describes the bicontinuous stability of STrIPS bijel fibers in a continuous oil phase. While prior research has primarily focused on the synthesis and applications of STrIPS bijels, this study provides for the first time results on their long-term stability. The bijels studied consist of two interwoven networks: one composed of water and glycerol, and the other of oils with varying compositions. These networks are stabilized by an interfacial layer of CTAB-functionalized Ludox TMA silica nanoparticles. We demonstrate control over bijel stability—ranging from hours to several weeks—by (i) enriching the surrounding oil with water and glycerol, (ii) preventing water evaporation, (iii) hydrophobizing the bijel support surface, and (iv) increasing the viscosity of the surrounding oil. These findings suggest that destabilization occurs through the loss of water and glycerol, which is counterbalanced by oil entering the bijel. Water and glycerol leave either through dissolution into the surrounding oil or wetting of the support surface, triggering the expansion of particle covered oil domains and slight shrinkage of the bijel fibers. The ability to control bijel stability, as established here, will be crucial for their application in fields such as energy storage, catalysis, and chemical separations.

## Author contributions

Meyer T. Alting: conceptualization, methodology, investigation, visualization, data curation, writing – original draft. Martin F. Haase: conceptualization, methodology, writing – original draft, supervision, project administration, funding acquisition.

## Data availabilty

The data supporting this article have been included as part of the ESI.†

## Conflicts of interest

The authors declare no competing financial interest.

## Supplementary Material

SM-021-D4SM01213E-s001

SM-021-D4SM01213E-s002

SM-021-D4SM01213E-s003
