# Supplementary material for: Stabilizing bicontinuous particle-stabilized emulsions formed via solvent transfer-induced phase separation
Source: Soft Matter. 2025 Jan 7;21(4):760–9. doi: 10.1039/d4sm01213e (PMC11721598; doi:10.1039/d4sm01213e)
Supplement: SM-021-D4SM01213E-s003 [file SM-021-D4SM01213E-s003.pdf]

Supplementary Information (SI) for Soft Matter.
This journal is © The Royal Society of Chemistry 2025

Supporting Information

Stabilizing Bicontinuous Particle-Stabilized Emulsions formed via Solvent Transfer-Induced Phase Separation

*Meyer T. Alting[1], Martin F. Haase[1]**

[1] Van ‘t Hoff Laboratory of Physical and Colloid Chemistry, Department of Chemistry, Debye Institute for Nanomaterials Science, Utrecht University, Utrecht, The Netherlands

* Correspondence: m.f.haase@uu.nl

This PDF file includes:

Supporting Information S1 CLSM image processing by ImageJ Fiji 2
Supporting Information S2 Determining oil-to-aqueous ratio σ and Σ via ImageJ Fiji 5
Supporting Information S3 Area-based and Volume-based Oil-to-Aqueous ratios 10
Supporting Information S4 Fit of σ for bijel in as-received *n*-dodecane....12
Supporting Information S5 Stabilization criterion of $\Sigma = 0.5$ 13
Supporting Information S6 Uncertainty in Stability Periods....14
Supporting Information S7 Aqueous content in bijel fibers....15
Supporting Information S8 Concentration of free water in oils....16
Supporting Information S9 Visual assessment of bijel stability of bijels in *n*-dodecane....17
Supporting Information S10 Stability of bijels in *n*-hexane and *n*-dodecane 20
Supporting Information S11 Hydrophilicity of CLSM container holding bijel fibers....22
Supporting Information S12 Viscosity of mixtures of *n*-dodecane in light mineral oil 23
Supporting Information S13 Change in oil-to-aqueous ratios for different oils 24
Supporting Information S14 Bijel stability cannot be purely described by diffusion 25
Supporting Information S15 Rate of destabilization of bijels in as-received oils....26
Supporting Information S16 Oil-to-aqueous ratio of water+glycerol enriched oils 27
Supporting Information S17 Physical properties of oils 28
Supporting Information S18 Movie captions....29
References 29

Supporting Information S1 CLSM image processing by ImageJ Fiji

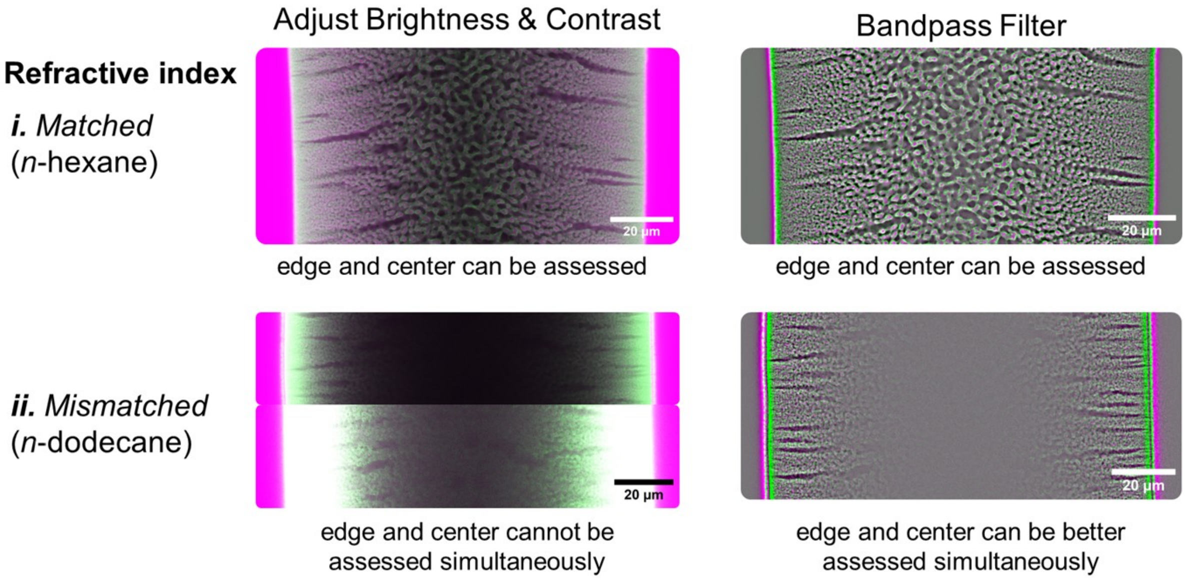

Figure S1A: CLSM images at the equator of bijel after image processing using (left column) adjusted brightness and contrast, or (right column) bandpass filter for bijels submerged in an oil with a (top row) matched and (bottom row) mismatched refractive index.

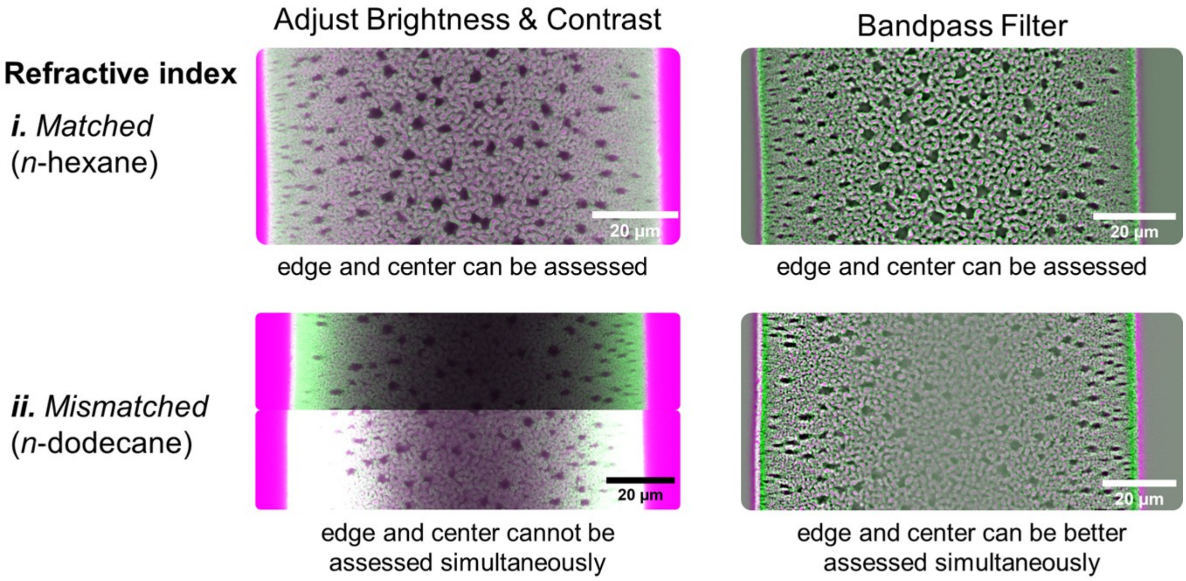

Figure S1B: CLSM images below the equator of the bijel fiber after image processing using (left column) adjusted brightness and contrast, or (right column) bandpass filter for bijels submerged in an oil with a (top row) matched and (bottom row) mismatched refractive index.

The bicontinuity of the bijels are assessed by confocal laser scanning microscopy (CLSM) at the equator of the bijel fiber. This technique relies on the matching the refractive index between the sample and its surroundings. *n*-Hexane has a similar refractive index to a bijel (1.36 versus 1.39, resp.), whereas toluene, *n*-dodecane and light mineral oil have higher refractive indices (resp. 1.497, 1.421 and 1.467). This mismatch limits the fluorescence signal detected originating from the center of the bijel fiber due to scattering.

Usually, we increase the image quality by adjusting the brightness and contrast of the CLSM image. Figure S1A shows the resulted CLSM image for both a bijel in a refractive index matched and mismatched medium. In case of matched refractive index, sufficient quality is obtained to assess the bicontinuity both at the center and near the edge of the fiber. Mismatch of the refractive index only allows imaging near the edge of the fiber. Oversaturating the CLSM detected fluorescence allows for imaging of the center of the bijel. However, the signal near the edge of the fiber is lost, limiting the bicontinuous assessment of the entire fiber within one frame.

Alternatively, applying a bandpass filter with autocorrection equalizes the signal intensity along the entire image. Hence, more signal can be obtained from the bijel's interior without oversaturating the CLSM image. This procedure relies on an algorithm based in Fourier space. ImageJ Fiji applies an bandpass filter by transforming the confocal image into Fourier space, removing the highest and lowest frequency signals that corresponds to the highest and lowest intensities. After that, it inverts the Fourier space back to real space. The resulting CLSM images are shown in the right column of Figure S1A.

The settings of the applied bandpass filter are: filter large structures: 40 px, filter small structures: 3 px, suppress stripes: none, tolerance of direction: 5%, autoscale after filtering and saturate image when autoscaling.

As the refractive index mismatch can still be too significant due to scattering to clearly assess the center of the fiber by CLSM, images below the equator have been acquired to assess the bicontinuity. As the path length shortens in this setting, less scattering occurs. Figure S1B shows the effect of the image quality of either adjusting brightness and contrast or applying a bandpass filter to the CLSM image. Determining the oil-to-aqueous area ratio between the center of the fiber or slightly below i.e. comparing Figures S1A and S1B hardly affected the oil-to-aqueous ratio.

Application of the bandpass filter removes the fluorescence signal outside the bijel. For clarification purposes, we want to keep the color around the fiber to be the false labeled magenta color as well, representative for the original CLSM image. This has been done by cropping the image to the fiber merge it by a magenta box as shown in **Figure S1C**. Furthermore, the bandpass filter limits the resolution near the edge of the fiber, hence we have not studied the structure close to the edge of the fiber. Therefore, our results in this manuscript are not affected by this color change applied.

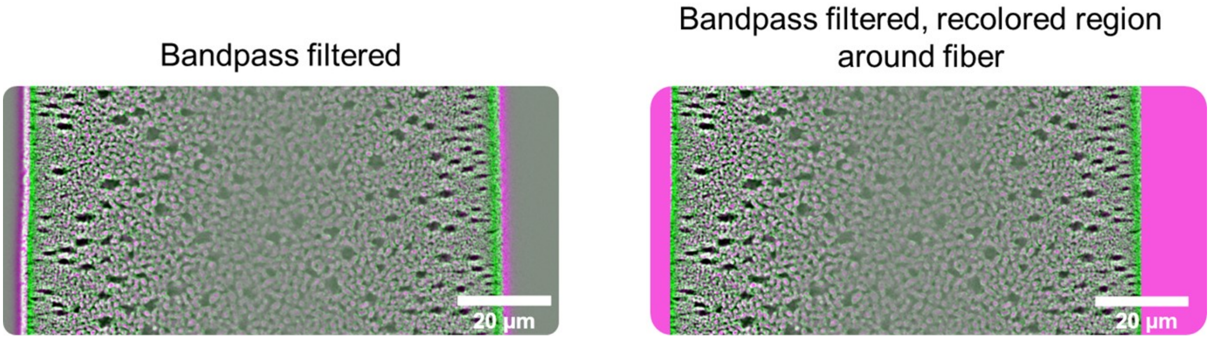

Figure S1C: CLSM images of a fiber cross section below the equator after applying a bandpass filter whereas the area around the fiber (left) is grey labeled due to the bandpass filter and (right) is replaced by magenta as representative with the original CLSM image.

Supporting Information S2 Determining oil-to-aqueous ratio σ and Σ via ImageJ Fiji

The oil-to-aqueous ratio (both for locally and overall bijel cross section, denoted by respectively σ and Σ) are estimated from CLSM images via image-processing in ImageJ Fiji. CLSM images acquired consist of the false magenta-labeled oil- and green-labeled particle channel. The oil-channel is used to determine the oil-signal in the bijel. The aqueous signal is estimated from the undetected signal from the oil- and particle merged image. The signals are based on the areas of oil- and aqueous phase in the bijel, approximated by thresholding the individual CLSM images. The following procedure has been applied, demonstrated for a bijel fiber stored in as-received *n*-dodecane after eight hours of storage.

Figure S2A shows the steps to obtain the threshold images needed for the oil- and aqueous channel. First, the bandpass filtered CLSM image is split into the individual oil- and particle channels. Both the individual oil-channel and a composite of the oil- and particle channel are converted into 8-bit image consisting of 256 colors, ranging in values between 0 and 255. A red-thresholding procedure has been applied to both images to divide the images into two-color images with values of only 0 (black) and 255 (white). The bandpass filter applied was: filter large structures: 20 px, filter small structures: 1 px, suppress stripes: none, tolerance of direction: 1% and autoscale after filtering.

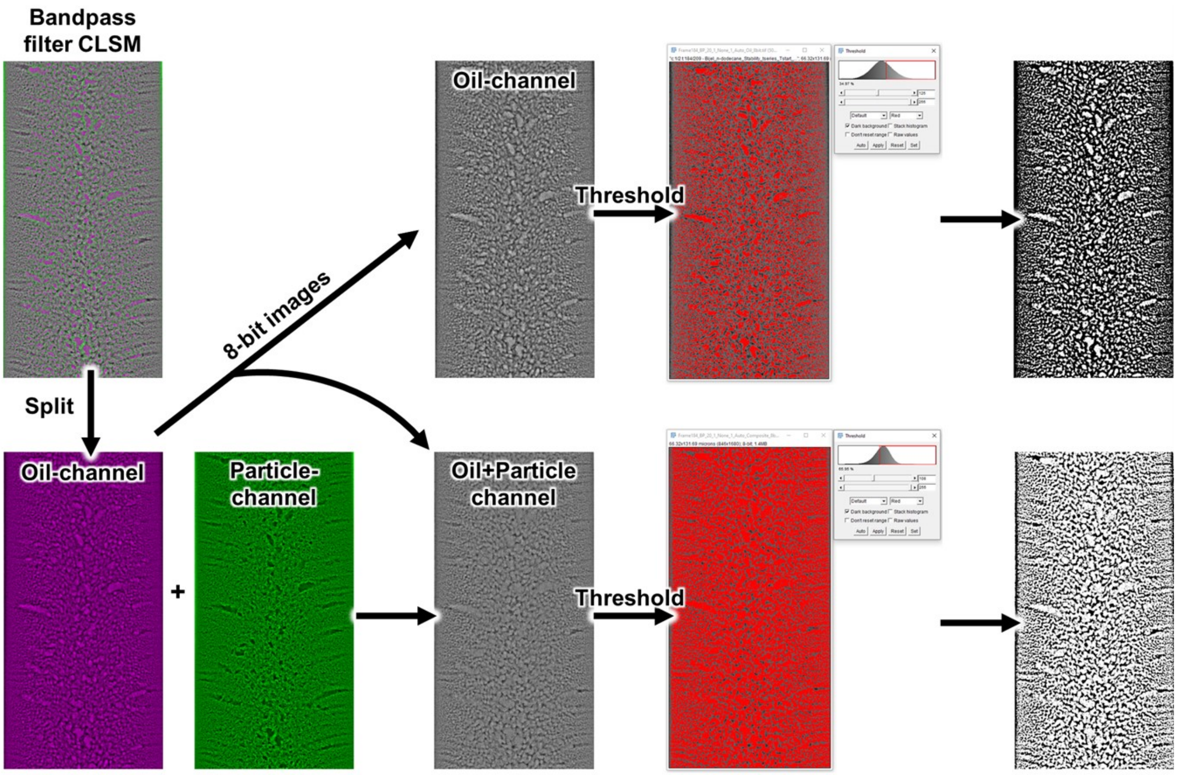

Figure S2A: Image-processing steps in splitting a bandpass-filtered confocal image into two threshold-applied images of the oil-only and oil and particle-merged channels.

The thresholding values are determined by comparing the CLSM-image with the 8-bit images as shown for an oil-channel in **Figure S2B**. Whereas the maximum threshold value is always kept at 255, the minimum value depends on the CLSM image (like mismatch in refractive index, laser intensity used, other practical differences). Comparing the red threshold pattern of the oil- and particles to the CLSM by naked eye allows to estimate the minimum threshold value for a proper mask.

When the threshold image seems to label too much or too less signal compared to the CLSM image, the minimum value was, respectively, raised or lowered. Varying this minimum has a significant effect on the oil-to-aqueous ratio. An example of improper assigning is shown in **Figure S2B**: comparing the red signal in the threshold oil-channel with the CLSM image shows that Σ ranges between 1.05 and 1.45. The threshold images, though, can be observed as being incorrect as the mask either leaves oil unlabeled or labels too much oil regions. Therefore, carefully inspecting the mask with the CLSM image is required.

As shown in the bottom figure of **Figure S2B**, a proper mask can be found at a minimum value of 131 which properly labels the oil in the CLSM image. The corresponding value for Σ equals 1.22. As the minimum value of the threshold can be rather close to the proper mask, the minimum value can vary by one or two points i.e. ranging between 129 and 133. **Figure S2C** shows the error ranges between 0.05 for stable and slightly destabilized structures and 0.10 for highly destabilized bijels. As the assessment of the CLSM images is done manually, some human error may be present in the data analysis.

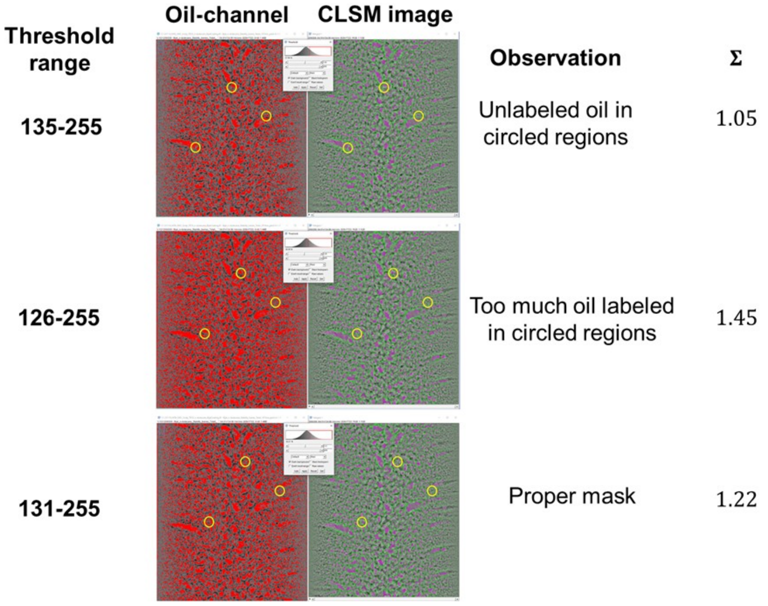

Figure S2B: Effect of varying the thresholding settings of the oil-channel on the oil-to-aqueous ratio in determining a proper threshold value. Improper assignation can be observed by comparing the threshold mask with the CLSM image.

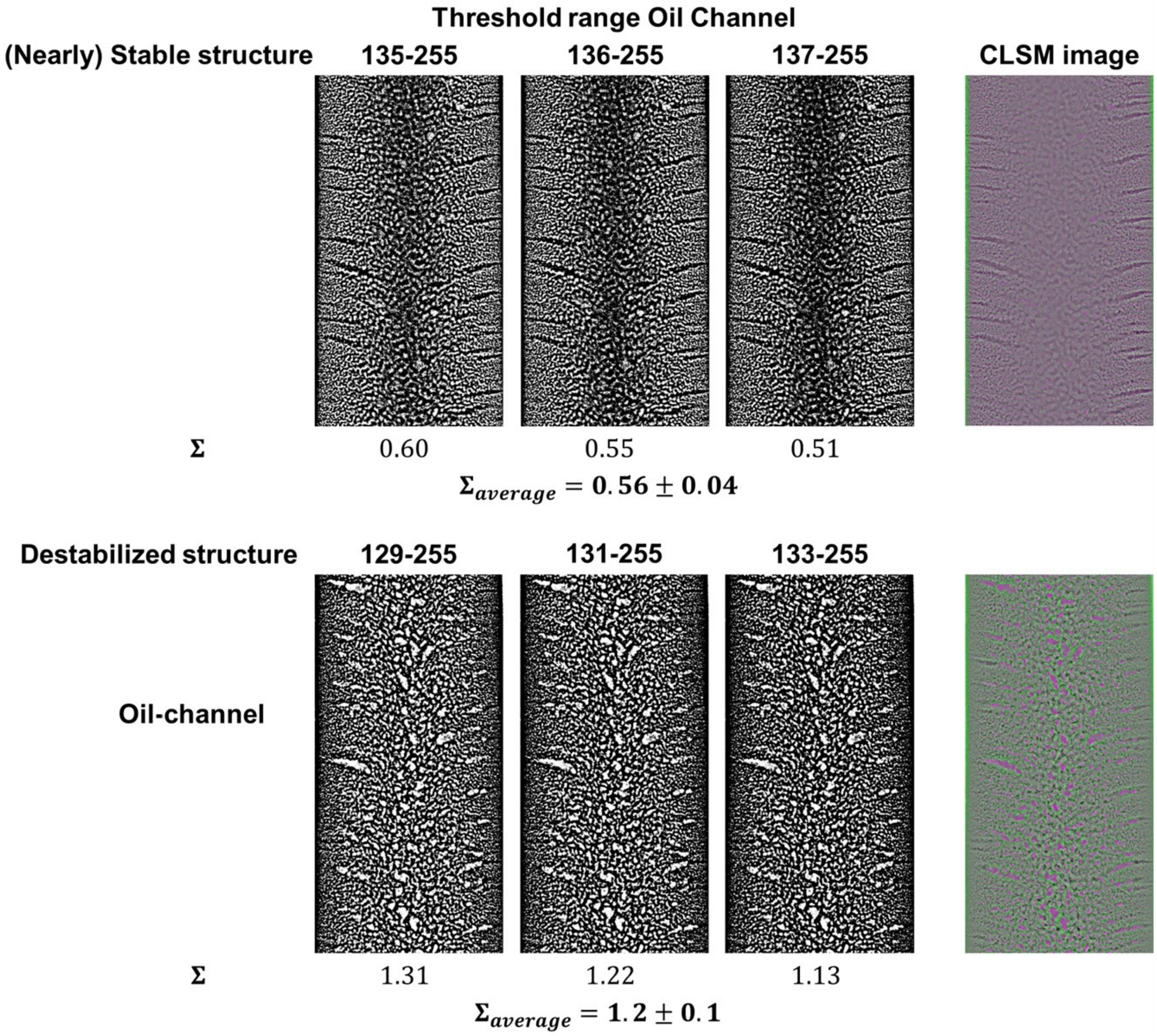

Figure S2C: Error in Σ after properly thresholding the oil-channel for (top row) stable and nearly stable bijel structure, and (bottom row) destabilized structure.

Practical differences like mismatches between the refractive index may affect the thresholding accuracy. As a result, the threshold values are often underestimated i.e. the actual oil- and aqueous areas are probably slightly higher than estimated. This effect, though, is often limited.

After thresholding the images, the oil- and aqueous areas can be determined. The oil-based area A_{oil} can be directly obtained from the threshold oil-image. To obtain the aqueous area, the oil+particle channel is inverted as shown in **Figure S2D**. This inversion labels the black-colored aqueous phase as white signal. As the areas around the fiber have to be excluded from the oil-to-aqueous ratio, the outer edges of the images are removed (if necessary). Last, the oil- and aqueous values (i.e. pixel value 255) are determined from the image's histogram. From this value, the overall oil-to-aqueous ratio Σ is determined.

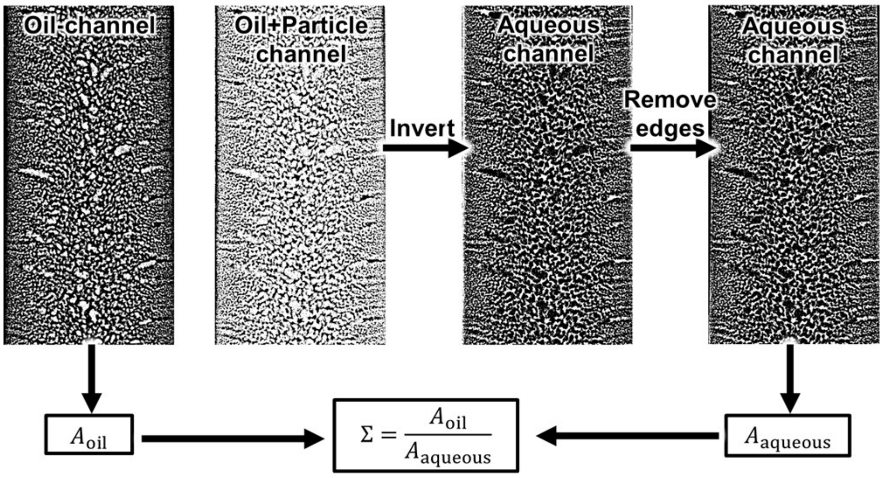

Figure S2D: Determining the oil and aqueous area from the threshold CLSM images via further image processing steps of the aqueous channel and how to determine the overall oil-to-aqueous ratio Σ of the bijel cross section.

To probe the local oil-to-aqueous ratio σ, thin bins are drawn along the length of the threshold-images of the fiber from **Figure S2D** as indicated in **Figure S2E**. Then, the oil-to-aqueous ratio for each bin at position r (center of bin) at r/r_0 is calculated i.e. σ among the different radial positions along the fiber.

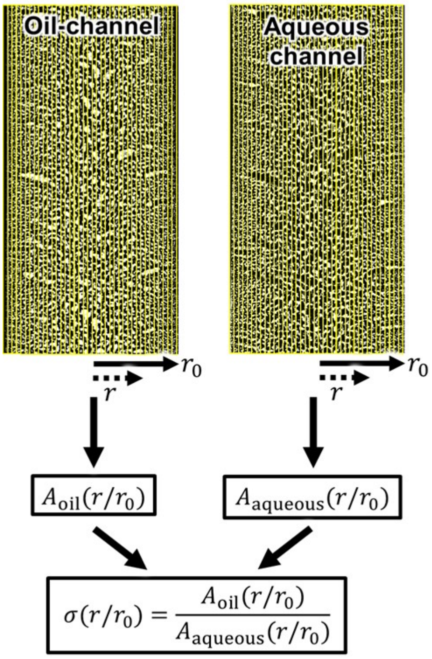

Figure S2E: Determining the local oil-to-aqueous ratio $\sigma(r/r_0)$ by binning the threshold CLSM images of the oil- and aqueous channel at various radial positions r/r_0.

Visualizing the change in oil-to-aqueous ratio is done by merging the threshold oil- and aqueous channel. **Figure S2F** shows the threshold images of oil and aqueous phase in a bijel stored in as-received *n*-dodecane. The oil is false labeled magenta, whereas the aqueous phase is labeled yellow. These images shows the clear change from aqueous-rich (yellow) to oil-rich after several hours. The actual values for both σ and Σ are plotted in **Figures 2C** and **2D** in the main text.

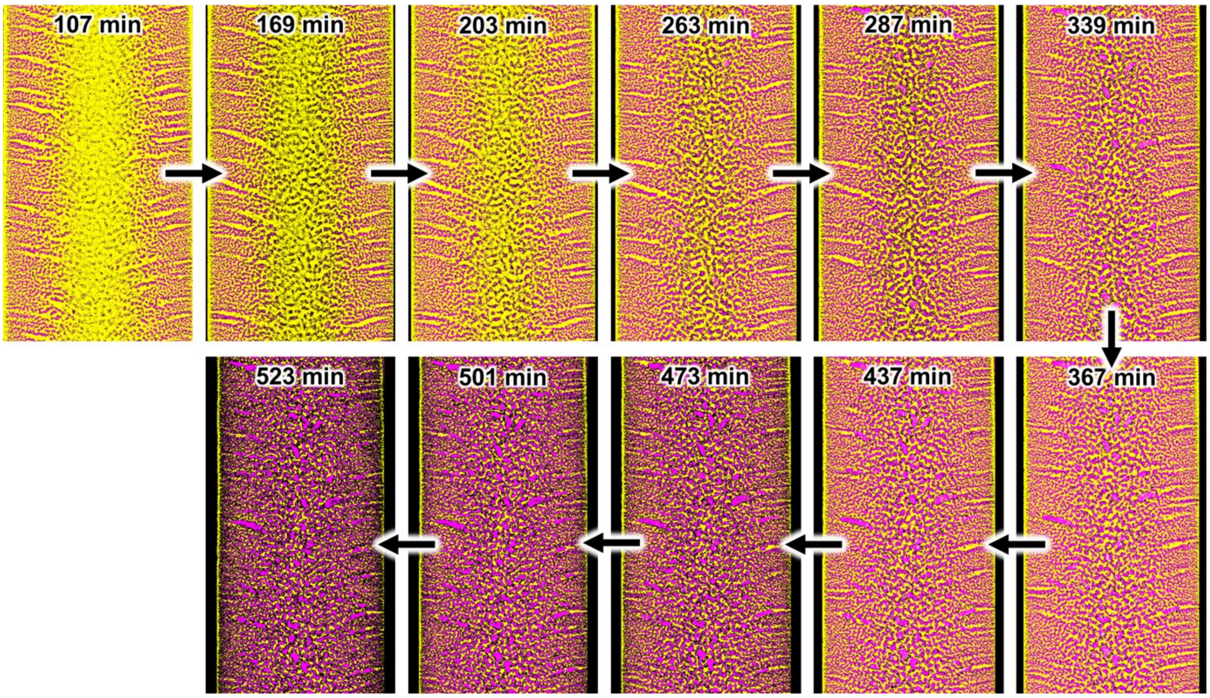

Figure S2F: Threshold confocal time series of oil- (magenta) and aqueous (yellow) channels for bijel fiber in as-received *n*-dodecane. The area around the fibers have been labeled black as it is not part of the fiber interior. The black area in the bijel fiber corresponds to particle signal, which are not taken into account in the oil-to-aqueous ratio assessment.

Supporting Information S3 Area-based and Volume-based Oil-to-Aqueous ratios

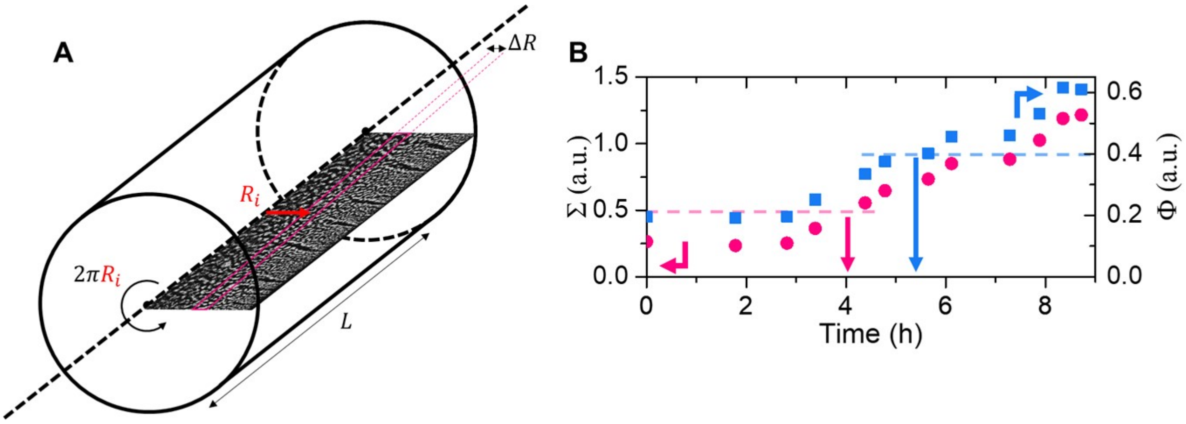

Figure S3: Calculation of volumetric oil-to-aqueous ratio Φ from area-based oil-to-aqueous ratio Σ. **A)** Sketch of 3D rotational symmetry used to calculate Φ. **B)** Plot of $\Sigma(t)$ (magenta, left *y*-axis) and $\Phi(t)$ (blue points, right *y*-axis) for a bijel stored in *n*-dodecane as studied in **Figure 2D** in the main text.

To account for oil/aqueous rearrangements three dimensions, the volumetric oil-to-aqueous ratio Φ can be estimated from the area-based oil-to-aqueous ratio Σ. Assuming a fiber being a cylinder with radius R and length L, applying rotational symmetry of CLSM images allows to estimate Φ similar as in our previous work (see SI section 10 and 11.2 in [12]). However, changes in the bijel may occur non-axisymmetric throughout the fiber due to e.g. gravity, which has not been imaged by CLSM. Therefore, applying axis-symmetry to 2D CLSM images can introduces large errors.

In brief, 2D CLSM images of oil- and aqueous channels (see **Figure S2E**) are divided in rectangular boxes with thickness ΔR and length L (see **Figure S3A**), located at radial position R_i with area $\Delta R{\cdot}L$. Rotating a box around the center axis forms a ring with volume $V_i = 2\pi{\cdot}R_i{\cdot}\Delta R{\cdot}L$.

The total volume of the oil- or aqueous phase corresponds to the sum of all volumes of the rings i.e.

$$V_{phase_i} = \sum_{i=1}^{n} 2\pi{\cdot}R_i{\cdot}\Delta R{\cdot}L$$

Then, Φ is calculated via $\Phi = \dfrac{V_{oil}}{V_{aqueous}}$.

Alternatively, the local area-based oil-to-aqueous ratio σ_i can be converted in the local volume-based oil-to-aqueous ratio ϕ_i using R_i, axis-symmetry and dividing it by the total circle area via

$$\phi_i = \frac{2\pi{\cdot}R_i{\cdot}\sigma_i}{\pi{\cdot}R^2}$$

(Note: the volume of the cylinder corresponds to $\pi \cdot R^2 \cdot L$, while σ_i corresponds to the average area of a liquid phase determined via $\sigma_i \propto \Delta R \cdot L$. Hence, no length is required in determining ϕ_i.)

Then, Φ can be calculated by summing over all ϕ_i via

$$\Phi = \sum_{i=1}^{n} \phi_i$$

Figure S3B shows $\Sigma(t)$ and $\Phi(t)$ for a bijel stored in *n*-dodecane (similar to **Figure 2** in the main text). This plot shows that both Σ and Φ remains constant for 3 hours and increases at longer times. Both plots have similar shapes, suggesting complementary evaluation irrespective of the method used.

The actual values for Σ and Φ differ significantly: Σ increases from 0.25 to 1.25 within 9 hours i.e. bijel changes from aqueous-rich into oil-rich. Φ increases from 0.2 to 0.6 within 9 hours, indicating that the bijel remained aqueous-rich. Besides, the bijel stability assessment is based on the time Σ doubles from 0.25 to 0.5. Analogously, Φ then double from 0.2 to 0.4. **Figure S3B** shows the stability of bijels corresponds to 4 hours (Σ) or 5 hours (Φ). The stability criteria of doubling Φ from 0.2 to 0.4, though, has not undergo critical evaluation; neither is the axisymmetric assumption valid.

Despite, the usage of the area-based stability criteria by evaluation Σ is valid in studying bijel destabilization kinetics. This method allows comparing bijels stored under different conditions.

Supporting Information S4 **Fit of σ for bijel in as-received *n*-dodecane**

The determination of σ from the thresholded CLSM images shows an increased spreading at longer times. To guide the eye through the plot, patterns of σ are fitted using a ninth-order polynomial fit. These raw and fitted patterns are shown in, respectively, **Figure S4A** and **S4B**.

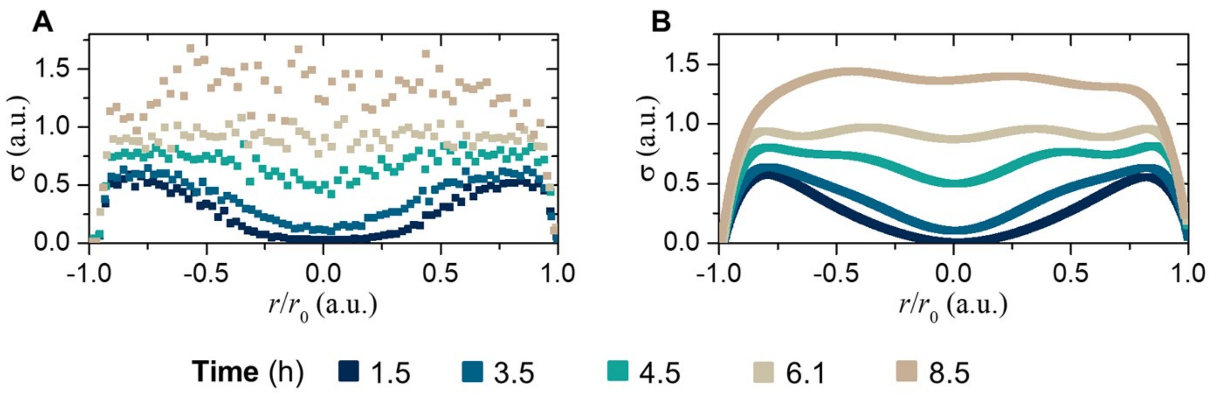

Figure S4: $\sigma(r/r_0)$ against r/r_0 for bijel fiber stored in as-received *n*-dodecane. **A)** Actual values for σ obtained from CLSM. **B)** Fitted patterns for σ using a ninth-order polynomial.

Supporting Information S5 Stabilization criterion of Σ = 0.5

To provide a rationale for our chosen bijel stability criterion of the area-based oil-to-aqueous ratio Σ = 0.5, we discuss the changes of Σ over time below. In **Figure S5** we analyze the increase of Σ due to two effects: I) leaving of the aqueous phase out of the bijel, and II) inflow and expansion of oil domains in the bijel structure.

I) The leaving of the aqueous phase is accompanied by a reduction of the fiber diameter (**Figure S5A**), and a decrease of the aqueous pore diameter (**Figure S5Bi**). Typically, both processes cause Σ to increase from 0.25 to 0.45 within 4 hours, because they reduce the overall area of the aqueous phase in the CLSM image.

II) The occurrence of oil domain expansion is observed from 4.4 hours on, as indicated by an arrow in **Figure S5A**. This oil expansion is quantified by the oil pore size over the normalized fiber radial position in **Figure S5Bii**. During the oil expansion, Σ increase to a value of 0.55 (**Figure S5A**), because the overall area of the oil phase increases in the CLSM image.

Across many different experimental conditions, Σ crosses the value of 0.5 in a similar manner, triggered by the expansion of oil domains. Thus, the crossing of Σ = 0.5 indicates the onset of oil flooding in the bijel fiber, our main criterion to compare bijel stability.

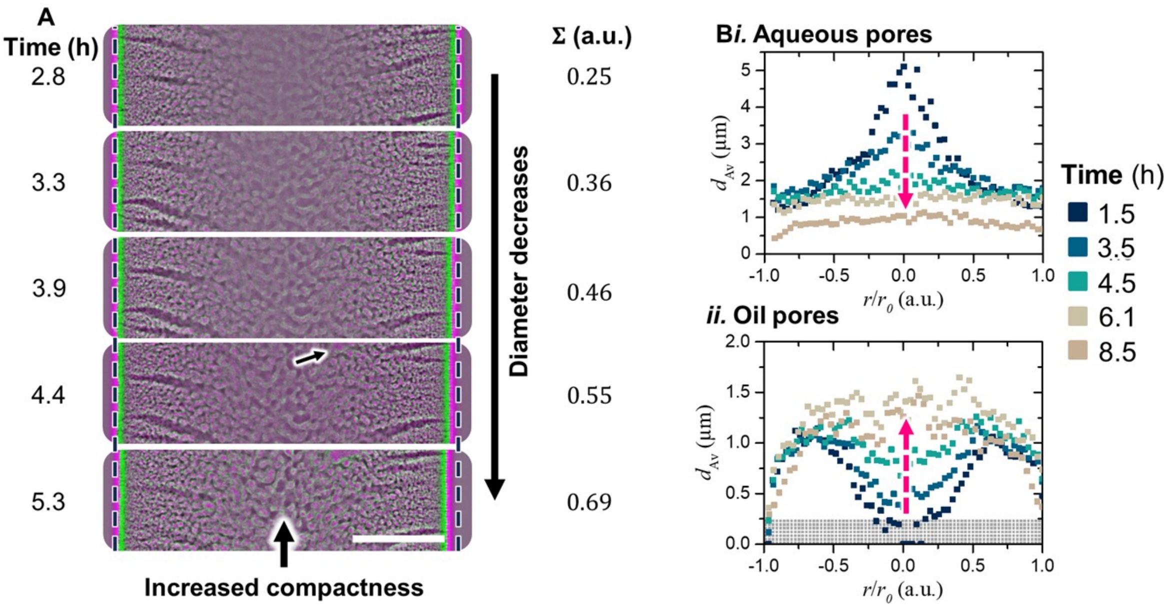

Figure S5: Effect of decrease of bijel fiber diameter on Σ for bijels stored in as-received *n*-dodecane. **A)** CLSM time series of bijel with Σ, dashed lines correspond to initial diameter of cross section. Scale bar is 20 µm. **B)** Sizes of ***i)*** oil pores and ***ii)*** aqueous pores in the bijel fiber at various times.

Supporting Information S6 Uncertainty in Stability Periods

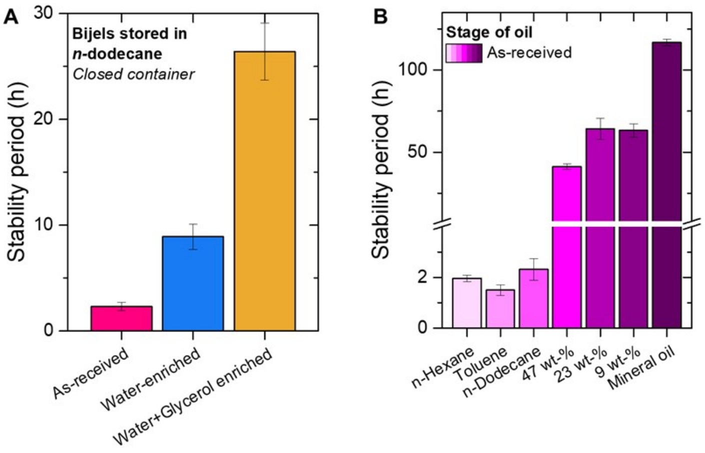

Figure S6: Stability periods of bijels stored in **A)** *n*-dodecane upon enrichment, and **B)** various oils by varying viscosity. These are the actual values including errors from the data present in **Figures 3A** and **5B** of the main text.

Figure S6 shows the stability period of bijels stored in various oils including the uncertainty in the measurement. These are the same stabilities as shown in **Figures 3A** and **5B** in the main text. The uncertainty is based on the times that Σ varies between 0.45 and 0.55 to comply with the error of the measurement i.e. Σ = 0.5 ± 0.05.

These plots show that the errors are relatively small compared to the actual data i.e. less than 15% deviation for *n*-dodecane. Although the error bars increase for *n*-dodecane upon longer stability periods as shown in **Figure S6A**, the relatively error decreases. As presented in the main text, the uncertainty in the stability periods reported are less than 15%.

Supporting Information S7 Aqueous content in bijel fibers

Commonly, around 5 cm of bijel fiber with a diameter of around 120 µm is stored in 2 mL of oil. The actual volumes of both water and glycerol in this fiber are estimated as follows.

Assuming a cylindrical geometry, the actual volume of a bijel fiber with length L and radius r is

$$V_{fiber} = \pi r^2 L = \pi \cdot \left(60\ \mu m * 10^{-3} \frac{mm}{\mu m}\right)^2 * \left(5\ cm * 10 \frac{mm}{cm}\right) = 0.57 mm^3 = 0.57\ \mu L$$

The volume fractions of all components present in the precursor including particles can be listed as $\varphi_{oil} = 0.070$, $\varphi_{water} = 0.387$, $\varphi_{glycerol} = 0.094$, $\varphi_{1-propanol} = 0.339$ and $\varphi_{TMA} = 0.110$ (values as previously reported in reference [1], whereas here the volume fraction of particles are included).

Assuming no volume shrinkage of the bijel fiber during STrIPS due to the inflow of oil, the same volume-fractions of water and glycerol are applying for the bijel fiber. This means that the actual volume of water in the bijel is

$$V_{water} = V_{fiber} \cdot \varphi_{water} = 0.57 \mu L \cdot 0.387 = 0.22\ \mu L$$

and similar for glycerol being

$$V_{glycerol} = V_{fiber} \cdot \varphi_{glycerol} = 0.57 \mu L \cdot 0.094 = 0.05\ \mu L$$

Since the bijel fiber is stored in 2 mL of *n*-dodecane and assuming all water and glycerol will dissolve in *n*-dodecane, the concentration of these components will be:

$$c_{water\ in\ n-dodecane} = \frac{V_{water}}{V_{n-dodecane}} = \frac{0.22\ \mu L}{2.0\ mL * 10^{-3} \frac{L}{mL}} = 109 \frac{\mu L_{water}}{L_{n-dodecane}}$$

$$c_{glycerol\ in\ n-dodecane} = \frac{V_{glycerol}}{V_{n-dodecane}} = \frac{0.05\ \mu L}{2.0\ mL * 10^{-3} \frac{L}{mL}} = 27 \frac{\mu L_{glycerol}}{L_{n-dodecane}}$$

Since both concentrations are below the maximum solubility of the respective component in *n*-dodecane (resp. 200 and 1600 µL/L), the entire aqueous phase can dissolve into the surrounding oil.

The solubility of glycerol in *n*-dodecane has been estimated from ref [2]. They reported a mutual solubility of ~0.005 mol glycerol per mol alkane (alkanes tested: *n*-pentane, *n*-hexane and *n*-heptane). As longer chains alkanes have similar polarity, we assumed that glycerol has similar solubilities in longer-chain hydrocarbons like *n*-dodecane. This means that a maximum solubility of 1.6 mL glycerol per liter

glycerol can be reached at 25 °C. The water solubility in *n*-dodecane, has been estimated to be similar to *n*-decane, as the actual concentration is still under debate for these long hydrocarbons.[3,4]

Supporting Information S8 **Concentration of free water in oils**

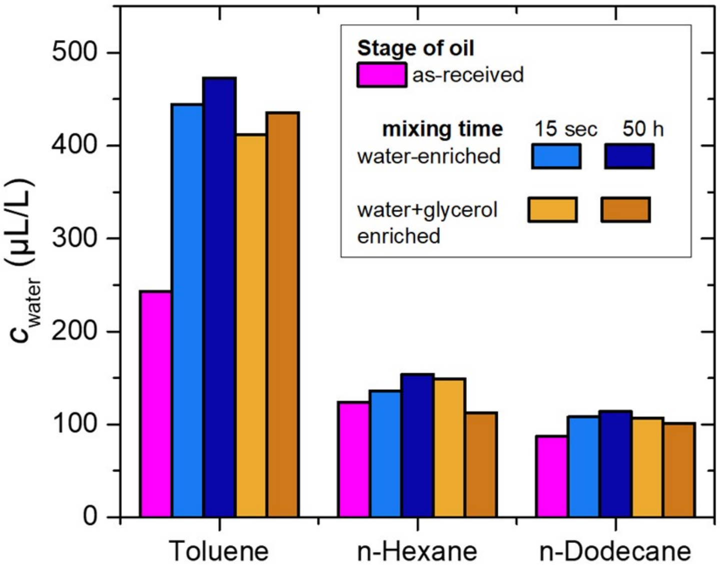

Figure S8: Concentration of free water in toluene, *n*-hexane and *n*-dodecane for different stages and various mixing times. The water-concentration was determined after phase separation the samples for 1 hour. The measurement error is 5 µL/L for all samples.

The concentration of free water is determined in as-received oils and enriched by water and both water and glycerol for 15 seconds or 50 hours at room temperature. The water concentration is high in toluene compared to *n*-hexane and *n*-dodecane due to the higher polarity of toluene. Mineral oil-based samples are not measured due to detection limitations. The experimental error may be larger than 5 µL/L as the glycerol concentration and its effect on the water concentration cannot been determined by our setup.

Enriching the oils by water-only increases the water concentration already within 15 seconds of mixing. Mixing it for longer times up to 50 hours increased the concentration further for all oils. This already showed that the longer mixing times increases the concentration of water further in the oils.

Interestingly, an opposite effect is observed for enriching alkanes by both water and glycerol. Mixing for 15 seconds increases the water concentration in all three oils, while mixing for 50 hours lowers the water concentration in *n*-hexane and *n*-dodecane. This decrease can be attributed to the slower dissolution of glycerol in alkanes compared to water. Mixing for longer times enables glycerol to slowly dissolve and phase separates part of the water due to a maximum uptake of both water and glycerol.

As all water-concentrations measured were below the maximum solubility, *n*-dodecane was heated to 60°C for 30 h while enriching by water and glycerol. The concentration increased to 109 µL/L when solely enriched by water, and slightly lowered to 105 µL/L when enriched by both water and glycerol. Since these values are still lower than the maximum solubilities, we concluded that simply heating the oil to increase the aqueous concentrations hardly affects the concentration of water.

Supporting Information S9 Visual assessment of bijel stability of bijels in *n*-dodecane

In the main text, the stability times of bijels have been assessed by evaluating the oil-to-aqueous area ratio Σ. Using this method, we gain time-resolved insights into the kinetics, stability and positional rearrangements of bijel structures. Additionally, it reveals that multiple/various destabilization mechanisms are at play affecting bijel stability.

Alternative methods to assess the stability of bijels do exist as well. One approach is via visually inspection of CLSM images by determining the first time at which the displacement of the aqueous phase by oil occurred in the bijel fiber.

Figure S9A shows CLSM time series of a cross section below the equator of a bijel fiber stored in water-enriched *n*-dodecane with covered container. The fiber showed no significant changes in structure within 8 h. After 22 h, several expanded oil-domains were observed. The bijel stability period is defined as the averaged time between the last stable and first destabilized structure. For this bijel fiber, the stability period is set to $\frac{8\,h + 22\,h}{2}$ = 14 h with an error of 8 h.

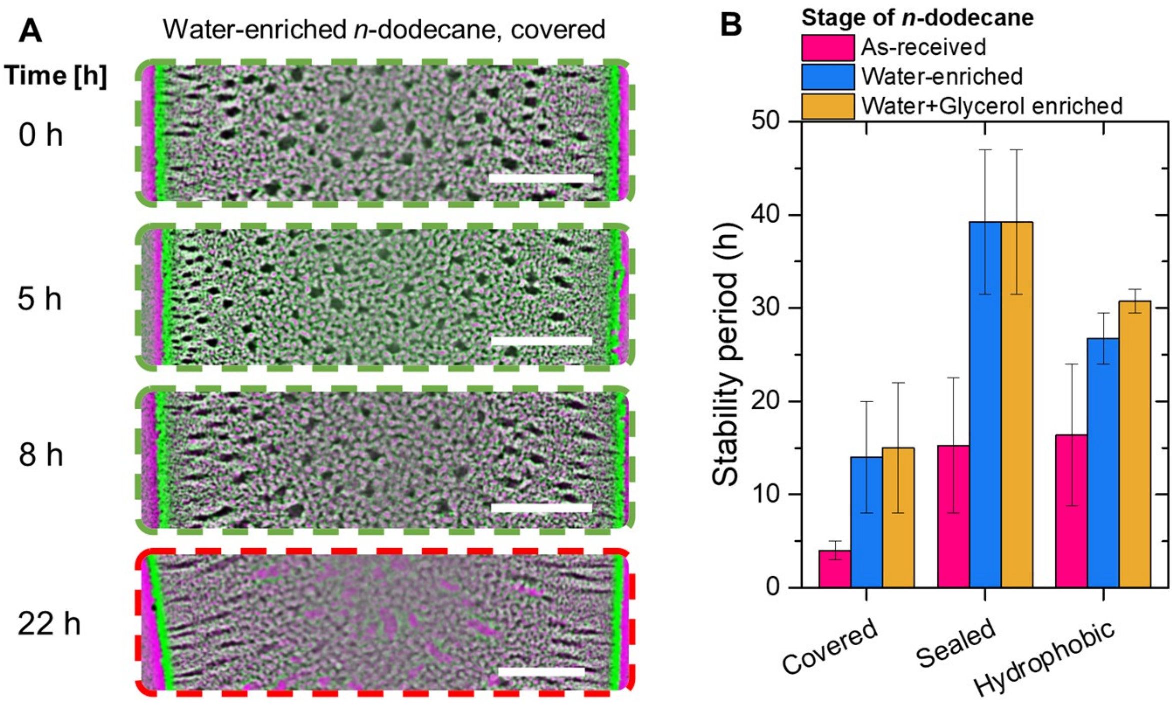

Figure S9: Visual assessment of bijel stability. **A)** CLSM time series of bijel fiber stored in water-enriched *n*-dodecane in covered hydrophilic glass. The green and red dashed borders indicate, respectively, stable and destabilized structures. **B)** Stability periods of bijel fibers stored in different states of *n*-dodecane in several types of containers.

Figure S9B plots the visual-based stability times of bijel fibers stored in *n*-dodecane either as-received, water-enriched or water+glycerol enriched. This plot shows that water- and water+glycerol enriched states have similar extents on the bijel stability. The usage of glycerol shows similar improvements based on the visual assessment method.

However, this visual approach cannot track the kinetics of oil/aqueous rearrangement as it does not consider the extent of destabilization. As a consequence, bijel fibers either containing limited number of oil expansions or that mostly displaced the aqueous phase by oil, can be labeled both as destabilized. Furthermore, the accuracy of this measurement depends on the time interval between image acquisition, introducing a large error when the structure destabilized during the night.

Figure S9C shows the stability periods as determined via the visual assessment and evaluation of Σ for bijel fibers stored in *n*-dodecane either as-received, water-enriched or enriched by both water and glycerol in a covered container. This plot shows similar bijel stabilities in as-received *n*-dodecane around 4 h via both methods. Upon enrichment by solely water, the stability period is ~15 h by visual inspection compared to 10 h as found by Σ. Enrichment by both water and glycerol results in stability times of 15 h (visual) and 25 h (Σ). The errors in the stability periods reported from Σ are significantly smaller than for the visual approach. The large discrepancies between both methods can be attributed to the different extents of oil/aqueous rearrangements which is only taken into account in Σ. These results shows that the stability period by evaluating Σ is more accurate than visual inspection.

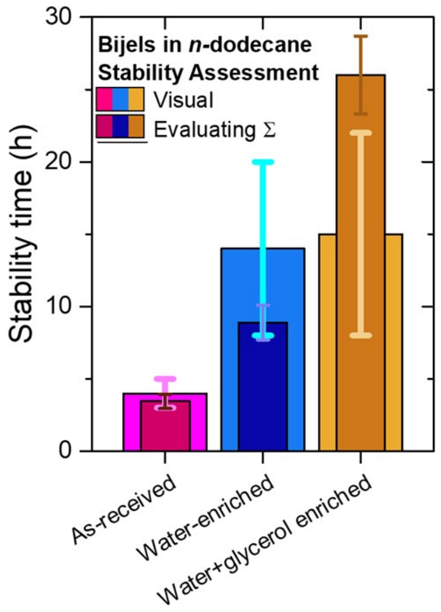

Figure S9C: Comparison of stability times for bijels stored in *n*-dodecane in a covered container as assessed by (bright colors) visual assessment of CLSM images, and (dark colors) evaluating Σ.

Figure S9B also shows the effect of evaporation as factor on the destabilization of the bijel. Roughly estimating the enhanced stability for glycerol-enriched *n*-dodecane compared to *n*-dodecane enriched solely by water showed only limited improvements. We hypothesized that, besides the dissolution of aqueous phase from the bijel into the surrounding oil, water may evaporate. The vapor pressure of *n*-dodecane, glycerol and water are, respectively, 0.018 kPa, 0.022 kPa and 3.1690 kPa. As water has significantly higher vapor pressure, it may easier evaporate in open containers at room temperature. This evaporation of water is significantly suppressed in the sealed container.

Supporting Information S10 Stability of bijels in *n*-hexane and *n*-dodecane

The stability of bijels depends on the viscosity of the oil and the enrichment of the surrounding oil by water and glycerol. However, the trends in stability for *n*-hexane and *n*-dodecane differs significantly. **Figure S10** shows the stability periods of bijel stored in *n*-hexane and *n*-dodecane, both as-received, water-enriched and water+glycerol enriched and stored in covered, hermetically sealed and hydrophobic containers.

As presented for *n*-dodecane, enriching the oil by water and glycerol improved the stability significantly as shown in **Figure S10B**. For *n*-hexane, though, it the stability of the bijel is less clear and depends on the container used. Storing in a hydrophilic, closed (covered) container showed that the stability of the bijel improved to 2 hours in enriched oils compared to as-received *n*-hexane. Interestingly, though, in a hydrophicic, sealed container, the stability for water-enriched *n*-hexane lowered to 1 hours, while with water+glycerol enrichement improved to around 15 hours. However, storing a hydrophobic, closed container, only the enrichment solely by water showed a significant enhanced stability; enriching by water and glycerol has a similar stability of bijels compared to storing in as-received *n*-hexane.

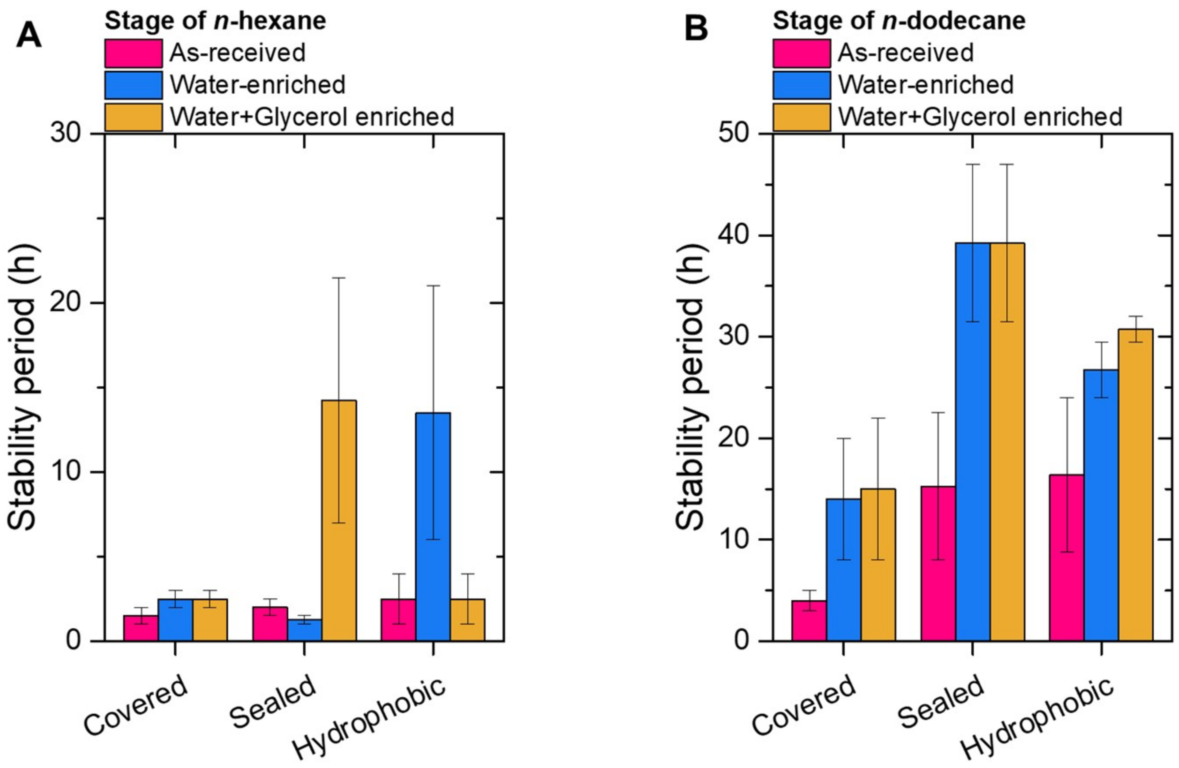

Figure S10: Stability periods of bijel fibers stored in different states of **A)** *n*-hexane and **B)** *n*-dodecane in several types of containers.

Besides the wetting effects of the glass surface and dissolution of the aqueous components, we also expect that evaporation of the oil plays a role here. Comparing the vapor pressures (p_i) of n-hexane, n-dodecane, water and glycerol at 25°C shows the following trend:

$$p_{n-hexane}\,(20.035\ kPa) > p_{water}\,(3.1690\ kPa) > p_{glycerol}\,(0.022\ kPa) > p_{n-dodecane}(0.018\ kPa)$$

This means n-hexane can evaporate easier than water and glycerol; the continuous oil phase will evaporate first. However, storing in n-dodecane will primary lead to water evaporation while keeping the continuous oil phase as liquid. This may then also affect the stability of bijels.

These differences in vapor pressures suggest that water evaporates from oils other than n-hexane. **Figures S10C** and **S10D** show containers filled with either a mixture of 9 wt-% n-dodecane in light mineral oil or n-hexane, respectively, after storing bijels for 24 h. The vials with n-dodecane/light mineral oil show water droplets on the inner walls. This indicates water-saturated air was present in the closed vial i.e. limited contact with surrounding air was possible. Interestingly, the vials filled with n-hexane were sealed and also showed some colorless liquid on the walls. We suggest that, by closing or sealing the containers by a coverslip, water-saturated air was formed. This may limit the further evaporation of water from the oils and so, from the bijels. Using water-saturated air in the containers may even further suppress the evaporation of water.

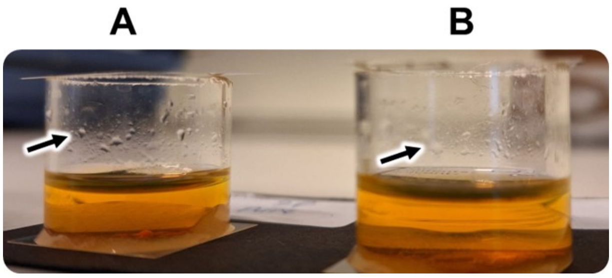

Figure S10C: Photographs of bijel fibers stored in a mixture of 9 wt-% n-dodecane in light mineral oil in **A)** as-received, and **B)** water+glycerol enriched oils in a closed container after 24 hours of storage.

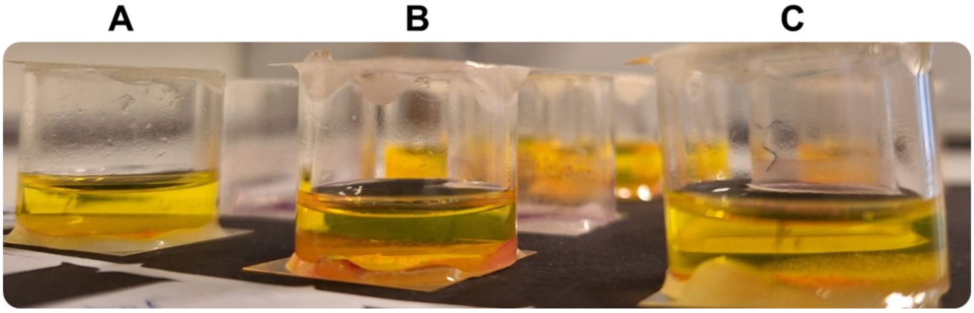

Figure S10D: Photographs of bijel fibers stored in *n*-hexane in **A)** as-received, **B)** water-enriched, and **C)** water+glycerol enriched oils in a sealed container after 24 hours of storage.

Supporting Information S11 Hydrophilicity of CLSM container holding bijel fibers

The cover glass of the CLSM containers holding the bijels are made from either Epredia or VWR, borosilicate glass. The glass surface is slightly hydrophilic: a sessile droplet of water in air shows a contact angle of around 45° for Epredia glass, while for VWR it is 55°. Treating the Epredia coverglass by OTS rendered the glass surface hydrophobic to contact angles of 149° as determined by a sessile water droplet in air as shown in **Figure S11**.

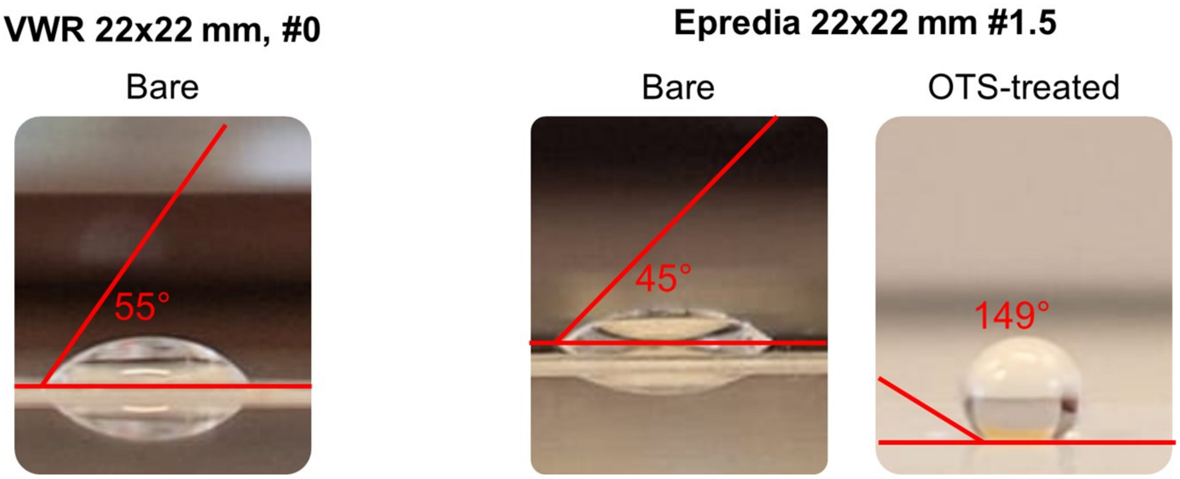

Figure S11: Contact angles of water sessile droplet in air on bare and OTS-treated coverslips.

Supporting Information S12 **Viscosity of mixtures of *n*-dodecane in light mineral oil**

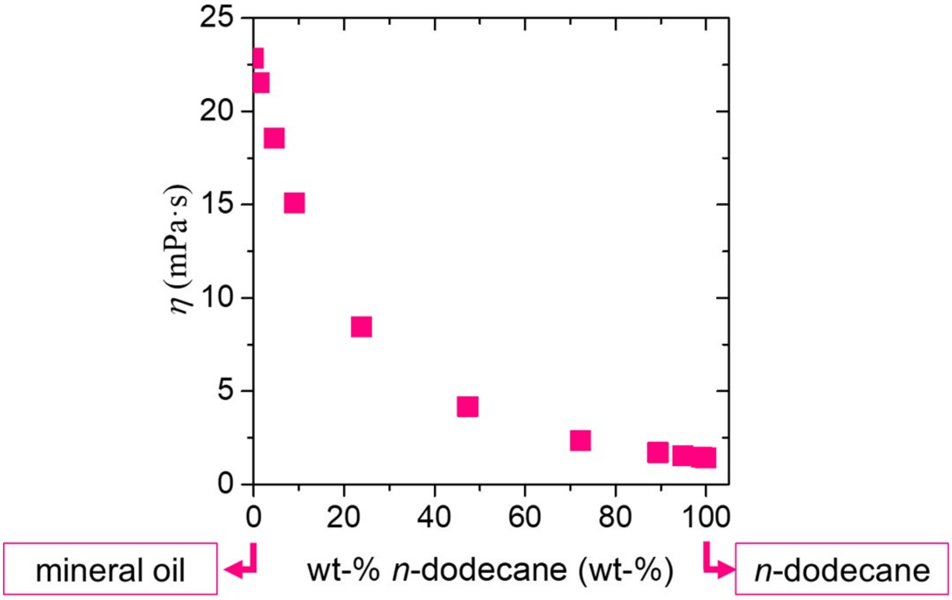

Figure S12: Viscosity of mixtures of light mineral oil plotted against the weight percentage of *n*-dodecane.

To obtain oil viscosities ranging between *n*-dodecane (1.395 mPa·s) and mineral oil (23.4 mPa·s), mixtures of both oils were prepared. **Figure S12** plots the viscosity of the mixtures between both oils against the wt-% of *n*-dodecane which is defined as $\frac{m_{n-dodecane}}{m_{n-dodecane} + m_{mineral\ oil}} \cdot 100\%$.

The mixtures containing 47, 23 and 9 wt-% *n*-dodecane were selected as mixtures with moderate viscosities between *n*-dodecane and mineral oil.

391 **Supporting Information S13 Change in oil-to-aqueous ratios for different oils**

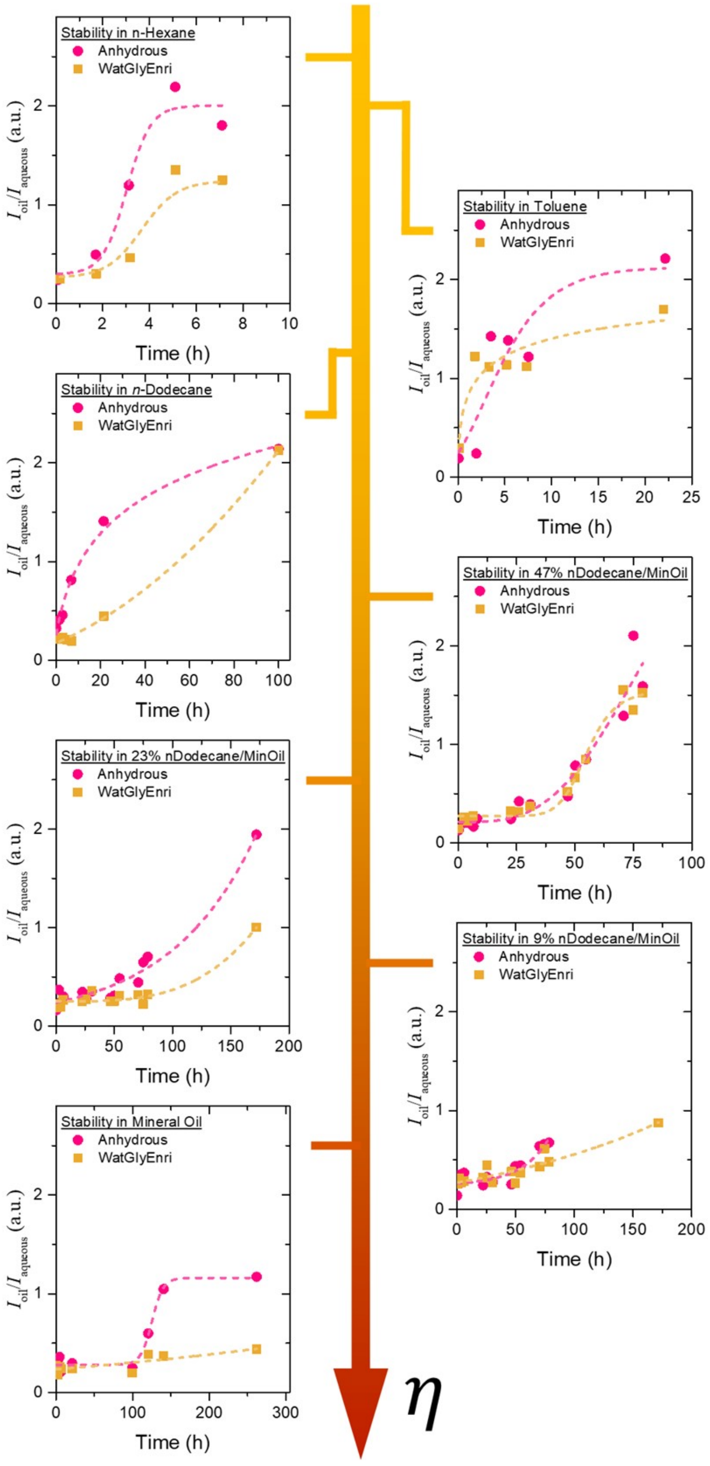

392

393 **Figure S13**: Plots of oil-to-aqueous ratio (corresponding to Σ) against time for all continuous oil phases
394 studied in this manuscript. These are used for oils in as-received (magenta, wrongly labeled as
395 anhydrous) and water+glycerol enriched (yellow, labeled as WatGlyEnri) stages, sorted for increasing
396 viscosity.

Supporting Information S14 **Bijel stability cannot be purely described by diffusion**

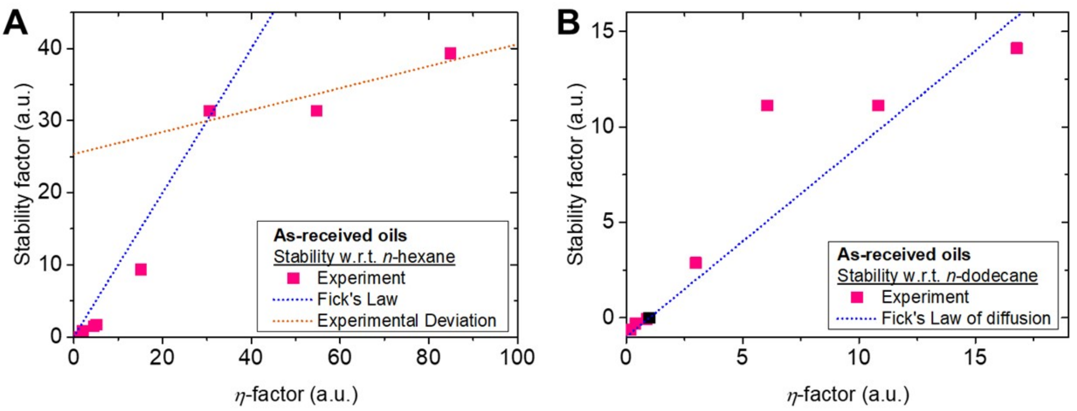

Figure S14: Stability factors against η-factors for all oils compared to **A)** *n*-hexane and **B)** *n*-dodecane. The blue dashed lines corresponds to the linear dependency as predicted by Fick's law of diffusion.

The effect of the viscosity of the surrounding oil on the rate of destabilization has been discussed in section 3.3 in the main text. To determine if this destabilization can be described by the leave of the aqueous phase by diffusion, we proposed a mathematical dependency of the stability time of the bijel related to Fick's law of diffusion. This can be derived as follows:

We hypothesize that the bijel stability depends inversely on the outward flux J of the aqueous phase i.e. stability $\sim J^{-1}$. This means that, for example, halving J will double the stability time. J is described by the diffusion coefficient D_i of the aqueous components and the concentration gradient ∇c_i via $J = -D_i \nabla c_i$. As the solubility of water and glycerol is similar in the alkanes studied, ∇c_i is similar for these oils. D_i depends inversely on the viscosity of the oil phase as described by Stokes law via $D_i \sim \eta^{-1}$.

This leads to the mathematical dependency that the stability time of the bijel is proportional to the viscosity of the oil i.e. stability $\sim \eta$ via stability $\sim J^{-1} \sim (D)^{-1} \sim (\eta^{-1})^{-1} = \eta$.

By determining the ratio of both the viscosity and stability with respect to a reference should reveal the extent of the diffusion of the aqueous leave on the bijel stability. **Figures S14A** and **S14B** shows the factor of the viscosity of all oils with respect to, respectively, *n*-hexane and *n*-dodecane. **Figure S14A** shows that up to increasing η by 30 times compared to *n*-hexane increases the stability by 30 times as well, as predicted by Fick's diffusion laws. Higher viscosities, then, reveals that the stability time is shorter than expected, which could reveal the presence of wetting effects of the glass. However, changing the reference to *n*-dodecane as shown in **Figure S14B** shows that only increasing η about 6

times higher than *n*-dodecane is not meeting Fick's law of diffusion. This shows that the interpretation depends on the reference take. Therefore, describing the stability time of the bijel via purely the change in viscosity is invalid.

Supporting Information S15 Rate of destabilization of bijels in as-received oils

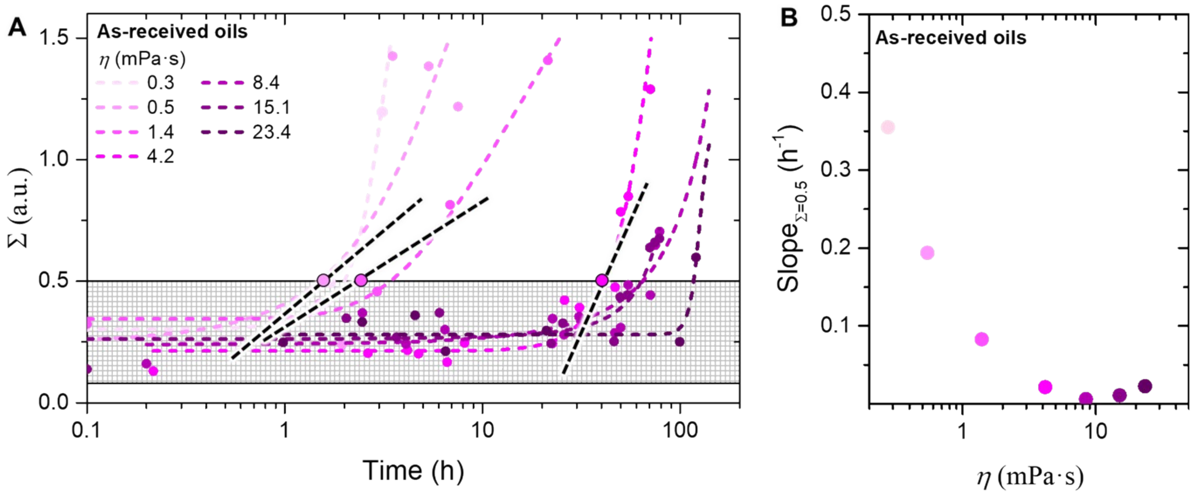

Figure S15: **A)** Plot of $\Sigma(t)$ for bijels stored in various as-received oils with different viscosities and indication in determing the rate of destabilization at $\Sigma = 0.5$ for all oils. **B)** Slope of $\Sigma(t)$ at $\Sigma = 0.5$ against viscosity.

Determining the slope at $\Sigma = 0.5$ shows the rate of destabilization as shown in **Figure S15A**. As can be seen, increasing the viscosity of the oil slows down the slope i.e. the rate of bicontinuity change. However, since these data has been assessed from the fitted data, the error is relatively big due to the type of fit used. Hence, the increased slope for viscosity values above 10 mPa·s may be misleading.

Also, due to the logarithmic time axis, the impression that the slope increases in **Figure S15A** for oil viscosities above 8.4 mPa·s, is invalid.

Supporting Information S16 Oil-to-aqueous ratio of water+glycerol enriched oils

Σ for all oils with various viscosities enriched by both water and glycerol have been determined and plotted in **Figure S16**. Compared to the as-received oils as shown in **Figure 5B** in the main text, the water+glycerol enriched oils have a less clear trend in rate of destabilization and stability period.

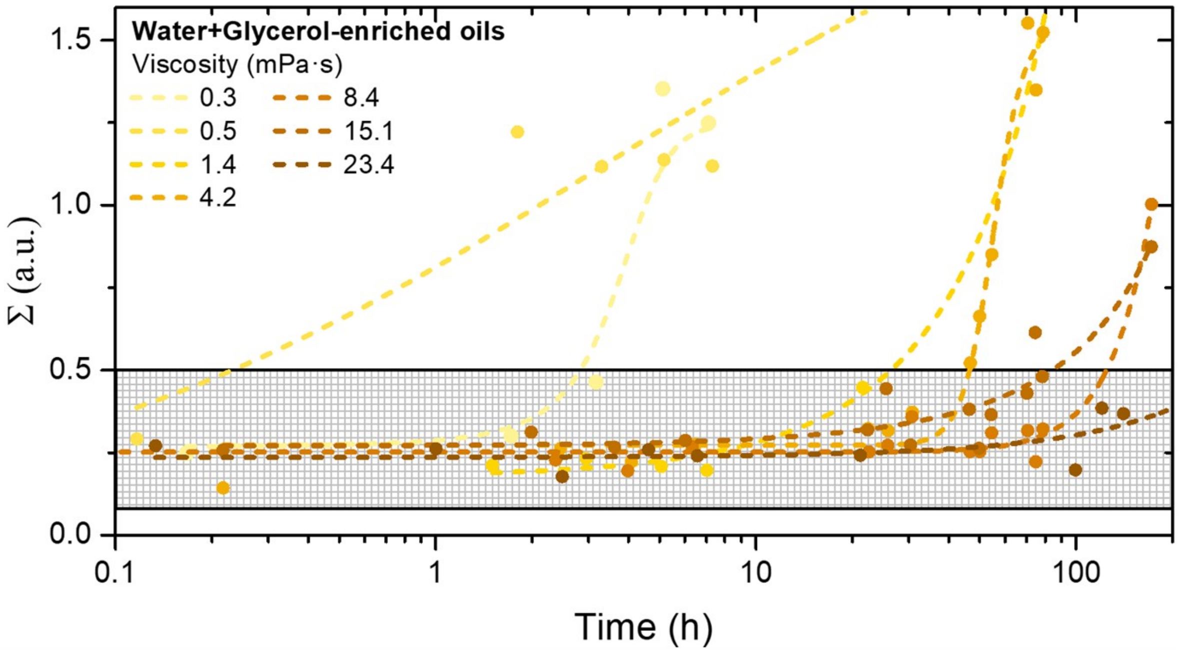

Figure S16: Effect of viscosity on bijel stability by storing in oils which are enriched by water and glycerol. Σ plotted on a logarithmic time axis, lines are drawn to guide the eye. The dashed grey rectangle highlights the region where the bijels are stable.

Supporting Information S17 Physical properties of oils

In table S17, the viscosity, dielectric constant, water solubility and interfacial tension for oil/water systems are listed.

Table S17: Viscosity, dielectric constant, water solubility and interfacial tension with water for some oils as used in the main text.

Oil	Viscosity^{a} [mPa·s]	Dielectric constant (at 25°C) [-]	Water solubility^{b} (at 25°C) [µL/L]	Interfacial tension [mN/m]
n-hexane	0.2758	1.89 [5]	65 [6]	50.8 [7]
toluene	0.5451	2.38 [5]	471 [8]	37.5 [9]
n-dodecane	1.3946	2.01 [5]	49 [3]	52.9 [7]
47 wt-% *n*-dodecane in mineral oil	4.1720	-	-	-
23 wt-% *n*-dodecane in mineral oil	8.4393	-	-	-
9 wt-% *n*-dodecane in mineral oil	15.0933	-	-	-
mineral oil	23.4044	2.31 [10] *at 50°C	-	34 [11] *surface tension, contact in air

a All viscosity values for these oils have been measured using the setup as mentioned in experimental section 2.9.

b Water solubility as reported in literature. These values, however, are under debate and can only be regarded as order of magnitude. Measurements performed for *n*-decane, for example, showed water solubilities ranging between 53 µL/L at 25°C (ref [3]) to 214 µL/L at 20°C (ref [4]). Therefore, except for toluene, we assume similar solubilities for all alkanes and mixtures of alkanes used in this study.

Supporting Information S18 Movie captions

Movie S1 (Separate file). CLSM time series of bijel fiber stored in as-received *n*-dodecane in a hydrophilic unsealed glass container processed via (left) adjusting brightness and contrast, or (right) applying a bandpass filter.

Movie S2 (Separate file). Videos of aqueous sessile droplets on OTS-treated Epredia coverslips stored in as-received, water-enriched and water+glycerol enriched *n*-dodecane. The aqueous droplet consists of either MilliQ pH 2 water or a 21 wt-% glycerol in MilliQ pH 2 water. 3.0 µL droplets felt for 1 mm on the substrate and moved along the glass surface. The droplets stack on the surface for enriched *n*-dodecane and slide on the surface for as-received *n*-dodecane without significant changes in the contact angle. This suggests that the interaction between the aqueous droplet and coverslip depends on the enrichment of the *n*-dodecane.

References

1 A. J. Sprockel, M. A. Khan, M. de Ruiter, M. T. Alting, K. A. Macmillan and M. F. Haase, *Colloids Surfaces A Physicochem. Eng. Asp.*, 2023, **666**, 131306.

2 A. E. Andreatta, A. Arposio, S. Ciparicci, M. B. Longo, F. Francescato, L. Gavotti and M. Fontanessi, *Congr. Argentino Ing. Quim.*

3 P. Schatzberg, *J. Phys. Chem.*, 1963, **67**, 776–779.

4 A. Becke and G. Quitzsch, *Chem.Techn.*, 1977, **29**, 49–51.

5 D. R. Lide, S. R. Data, E. A. Board, G. Baysinger, S. Chemistry, C. E. Library, L. I. Berger, R. N. Goldberg, B. Division, H. V Kehiaian, K. Kuchitsu, G. Rosenblatt, D. L. Roth and D. Zwillinger, in *CRC Handbook of Chemistry and Physics*, CRC Press, Boca Raton, FL, 2005, pp. 15.16-15.25.

6 J. W. Roddy and C. F. Coleman, *Talanta*, 1968, **15**, 1281–1286.

7 S. Zeppieri, J. Rodríguez and A. L. López De Ramos, *J. Chem. Eng. Data*, 2001, **46**, 1086–1088.

8 P. K. Glasoe and S. D. Schultz, *J. Chem. Eng. Data*, 1972, **17**, 66–68.

9 J. Saien and S. Akbari, *J. Chem. Eng. Data*, 2006, **51**, 1832–1835.

10 V. M. Shkol'nikov, L. A. Bronshtein, Y. N. Shekhter and O. L. Drozdova, *Chem. Technol. Fuels Oils*, 1977, **7**, 479–481.

11 S. Bashir, X. C. I. Solvas, M. Bashir, J. M. Rees and W. B. J. Zimmerman, *Biochip J.*, 2014, **8**, 122–128.

12 M. A. Khan, A. J. Sprockel, K. A. Macmillan, M. T. Alting, S. P. Kharal, S. Boakye-Ansah and M. F. Haase, *Adv. Mater.*, 2022, **34**, 2109547.
